# Supplementary figures and images for: A multiscale model of complex endothelial cell dynamics in early angiogenesis
Source: PLoS Comput Biol. 2021 Jan 7;17(1):e1008055. doi: 10.1371/journal.pcbi.1008055 (PMC7817011; doi:10.1371/journal.pcbi.1008055)

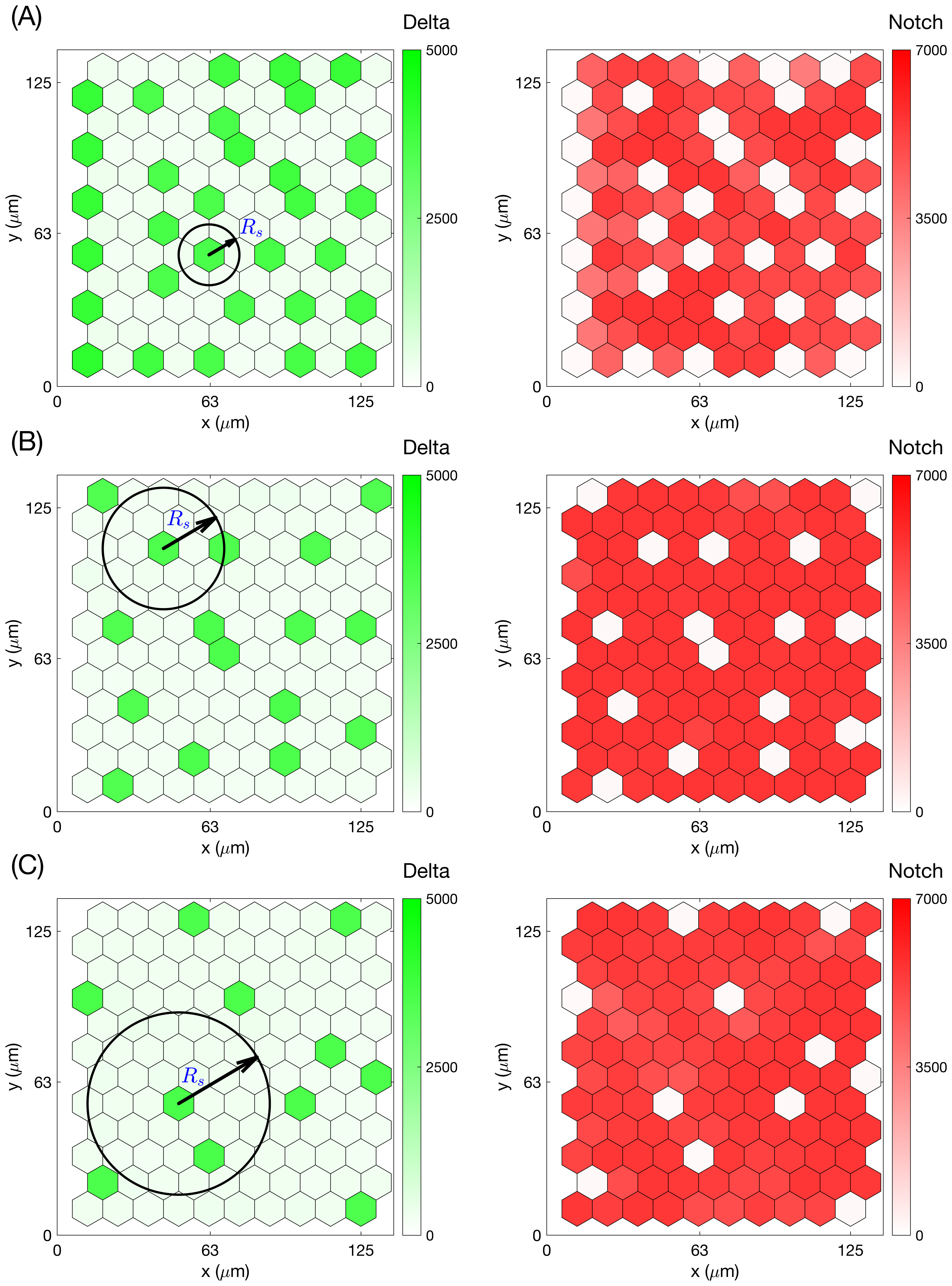

Supplement: S1 Fig — Final steady state patterns established during single stochastic simulations of the system described by the kinetic reactions outlined in Fig 3D for a uniform hexagonal lattice of 10 × 12 voxels. (A) Rs = 1.0h, (B) Rs = 2.0h, (C) Rs = 3.0h and the rest of the parameter values as in S1 Table. (TIF) [file pcbi.1008055.s002.tif]

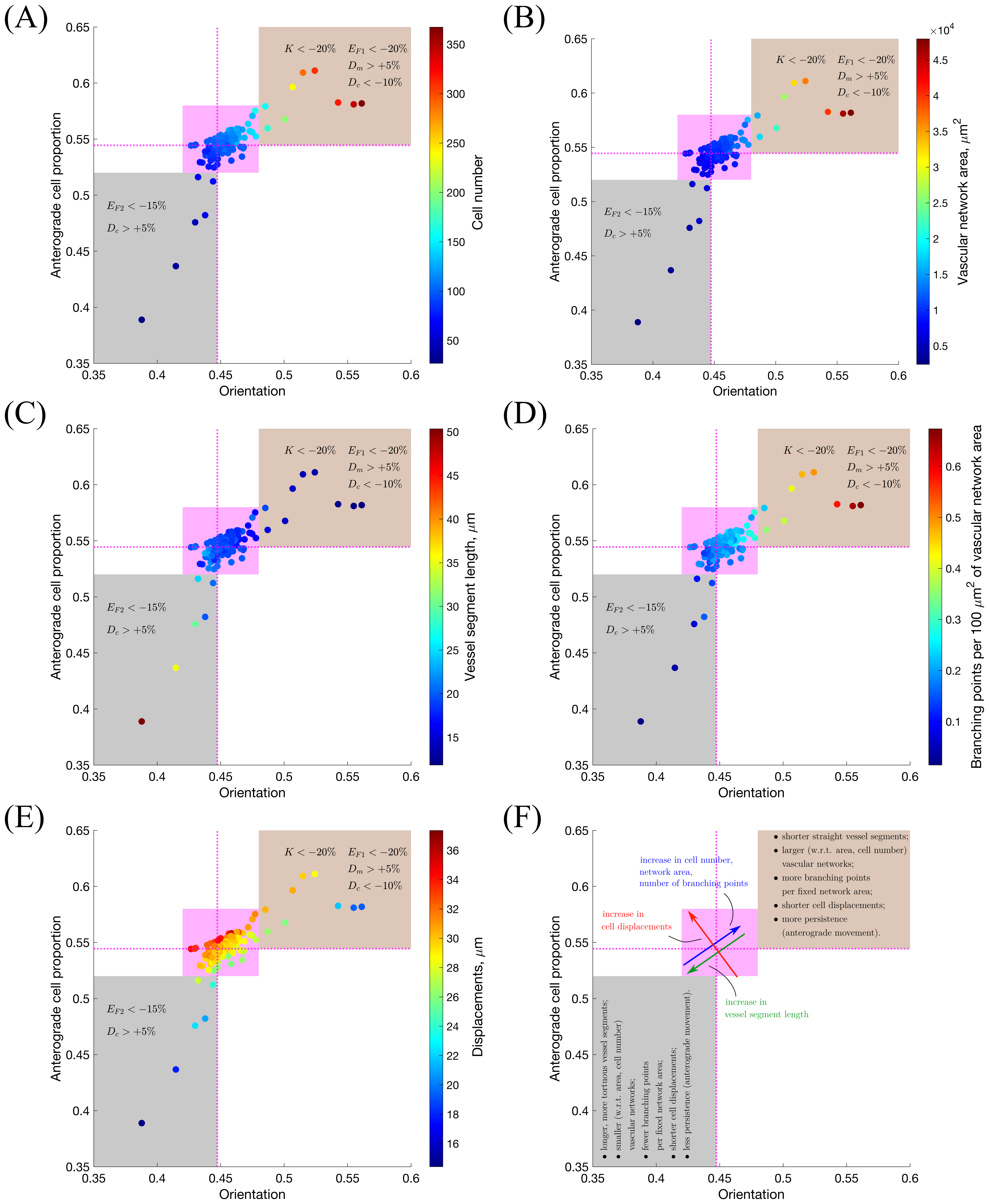

Supplement: S2 Fig — We performed simulations of our model by varying one of the parameters of the cellular and tissue scales at a time by a fixed per cent and keeping default values for the rest of the parameters (as in S2 Table). Each parameter was varied by ±0.1%, ±1%, ±5%, ±10%, ±15% and ±20%. For each numerical experiment, several quantitative metrics were computed. The results are represented as scatter plots of mean cell trajectory orientation vs. mean anterograde cell proportion with colouring indicating mean (A) cell number; (B) vascular network area; (C) vessel segment length; (D) number of branching points per 100 μm2 of vascular network area and (E) displacements. On these plots, dashed magenta lines indicate the point corresponding to the default parameter values; magenta highlights the region of the main point clustering. The grey-coloured outlier region corresponds to vascular networks with less persistent, twisted vessels, whereas the brown outlier region is characterised by longer straight vessel segments. Variations of the parameters that push the system towards one of the outlier regions are indicated on each plot. Panel (F) provides a general summary of these results. Simulation setup as in Setup 1, S3 Table, with Tmax = 2.5. The results are averaged over 100 realisations. The subcellular parameters were fixed at their default values in all experiments (see S1 Table). (TIF) [file pcbi.1008055.s003.tif]

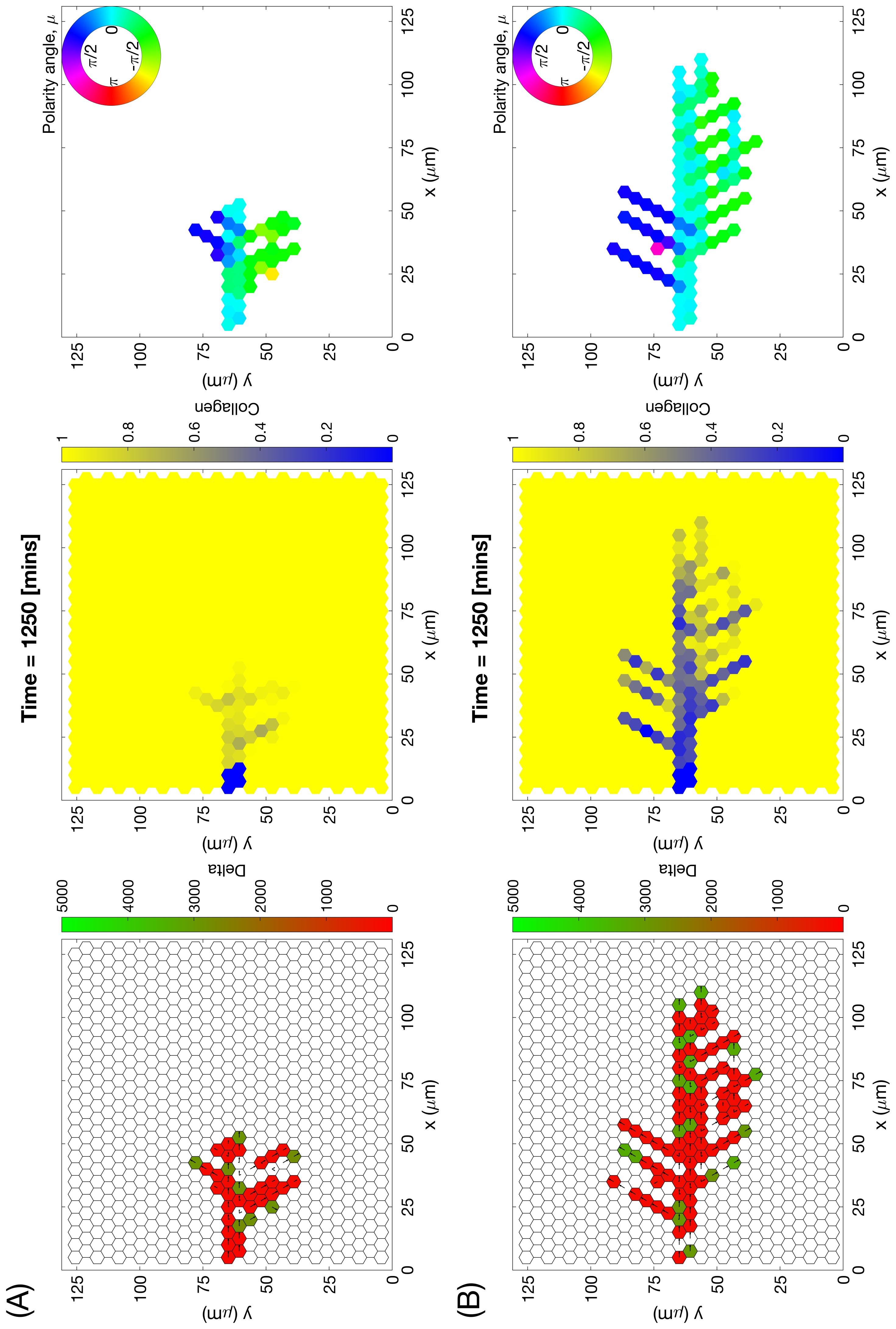

Supplement: S3 Fig — Final configurations of simulated vascular networks of VEGFR2+/- mutant cells growing in uniform concentration of VEGF = 5 ng/ml, plot (A), and VEGF = 50 ng/ml, plot (B). The leftmost panels show the amount of Delta, D. Higher values (green colour) correspond to tip cell phenotype, low values (red colour)—to stalk. On these plots arrows correspond to the orientation landscape configuration, l. The central panels indicate the final concentration of the ECM, c. The rightmost panels—final distribution of the mean polarity angle, μ, variable. Numerical simulations were performed using Setup 1 from S4 Table and Tmax = 2.5. Parameter values are listed in S1 and S2 Tables for subcellular and cellular/tissue scales, respectively, except of those changed for VEGFR2+/- mutant cells (see S1 Appendix). (TIF) [file pcbi.1008055.s004.tif]

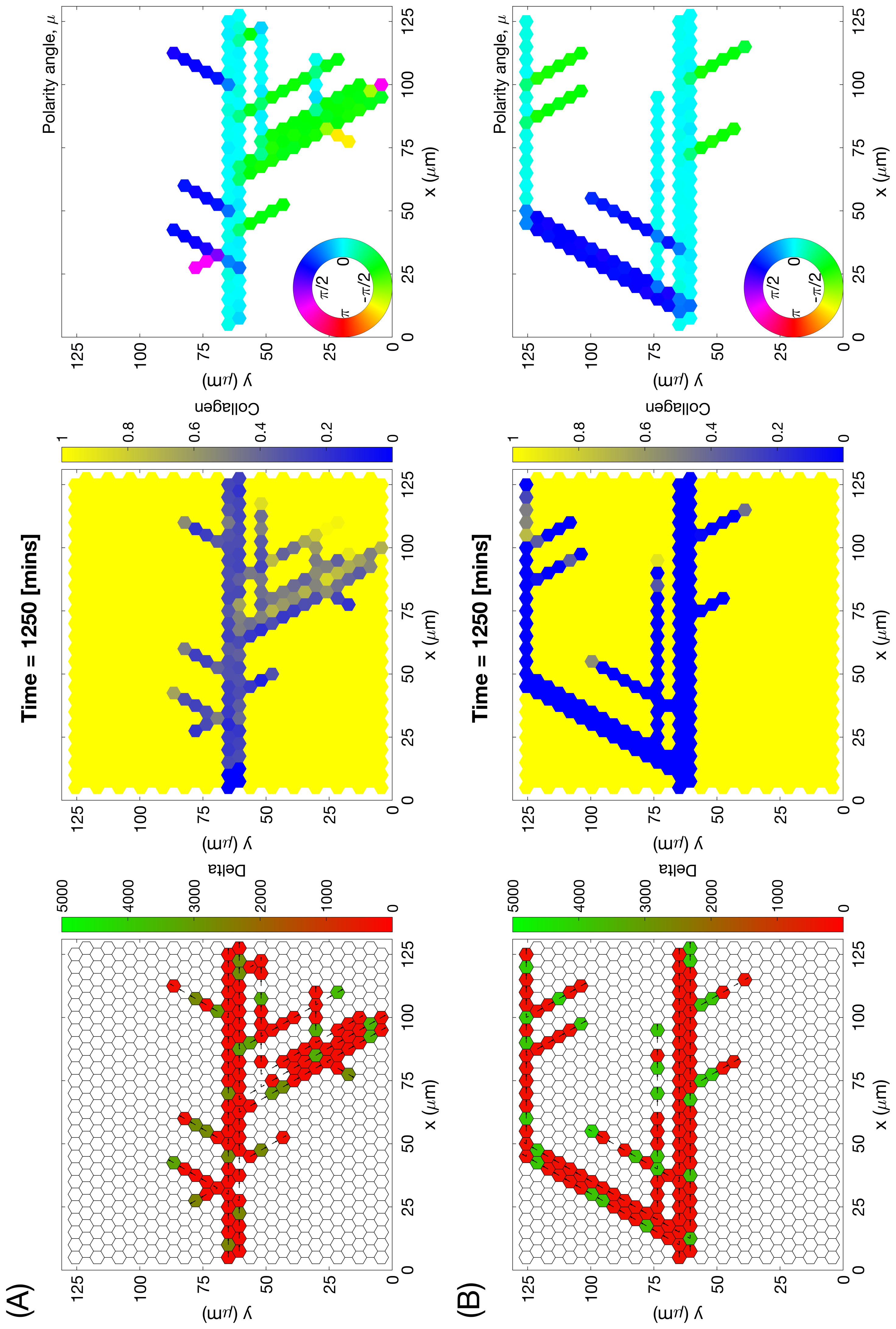

Supplement: S4 Fig — Final configurations of simulated vascular networks of VEGFR1+/- mutant cells growing in uniform concentration of VEGF = 5 ng/ml, plot (A), and VEGF = 50 ng/ml, plot (B). The leftmost panels show the amount of Delta, D. Higher values (green colour) correspond to tip cell phenotype, low values (red colour)—to stalk. On these plots arrows correspond to the orientation landscape configuration, l. The central panels indicate the final concentration of the ECM, c. The rightmost panels—final distribution of the mean polarity angle, μ, variable. Numerical simulations were performed using Setup 1 from S4 Table and Tmax = 2.5. Parameter values are listed in S1 and S2 Tables for subcellular and cellular/tissue scales, respectively, except of those changed for VEGFR1+/- mutant cells (see S1 Appendix). (TIF) [file pcbi.1008055.s005.tif]

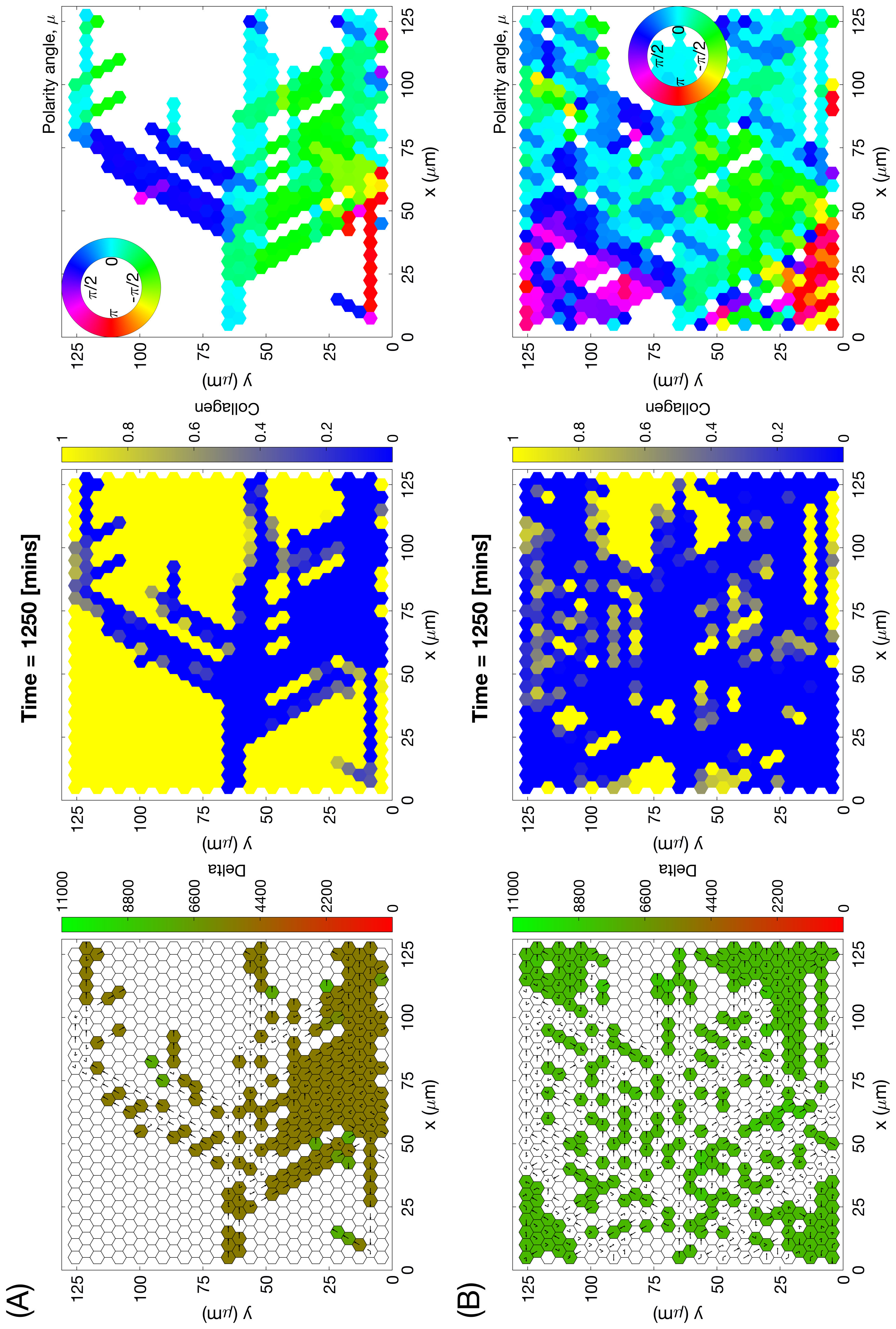

Supplement: S5 Fig — Final configurations of simulated vascular networks of VEGFR2+/- mutant cells treated with DAPT growing in uniform concentration of VEGF = 5 ng/ml, plot (A), and VEGF = 50 ng/ml, plot (B). The leftmost panels show the amount of Delta, D. Higher values (green colour) correspond to tip cell phenotype, low values (red colour)—to stalk. On these plots arrows correspond to the orientation landscape configuration, l. The central panels indicate the final concentration of the ECM, c. The rightmost panels—final distribution of the mean polarity angle, μ, variable. Numerical simulations were performed using Setup 1 from S4 Table and Tmax = 2.5. Parameter values are listed in S1 and S2 Tables for subcellular and cellular/tissue scales, respectively, except of those changed for VEGFR2+/- mutant cells and DAPT treatment (see S1 Appendix). (TIF) [file pcbi.1008055.s006.tif]

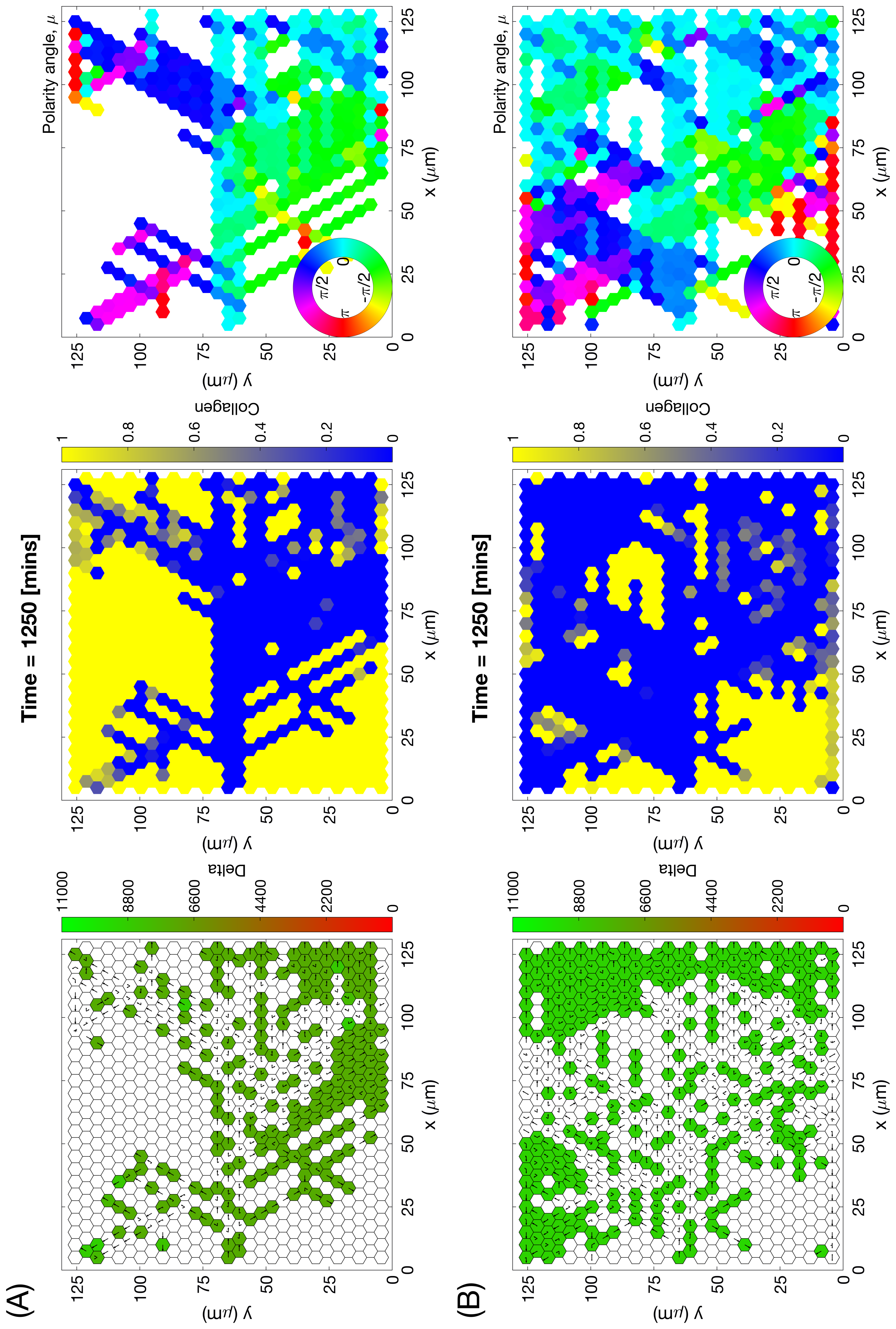

Supplement: S6 Fig — Final configurations of simulated vascular networks of VEGFR1+/- mutant cells treated with DAPT growing in uniform concentration of VEGF = 5 ng/ml, plot (A), and VEGF = 50 ng/ml, plot (B). The leftmost panels show the amount of Delta, D. Higher values (green colour) correspond to tip cell phenotype, low values (red colour)—to stalk. On these plots arrows correspond to the orientation landscape configuration, l. The central panels indicate the final concentration of the ECM, c. The rightmost panels—final distribution of the mean polarity angle, μ, variable. Numerical simulations were performed using Setup 1 from S4 Table and Tmax = 2.5. Parameter values are listed in S1 and S2 Tables for subcellular and cellular/tissue scales, respectively, except of those changed for VEGFR1+/- mutant cells and DAPT treatment (see S1 Appendix). (TIF) [file pcbi.1008055.s007.tif]

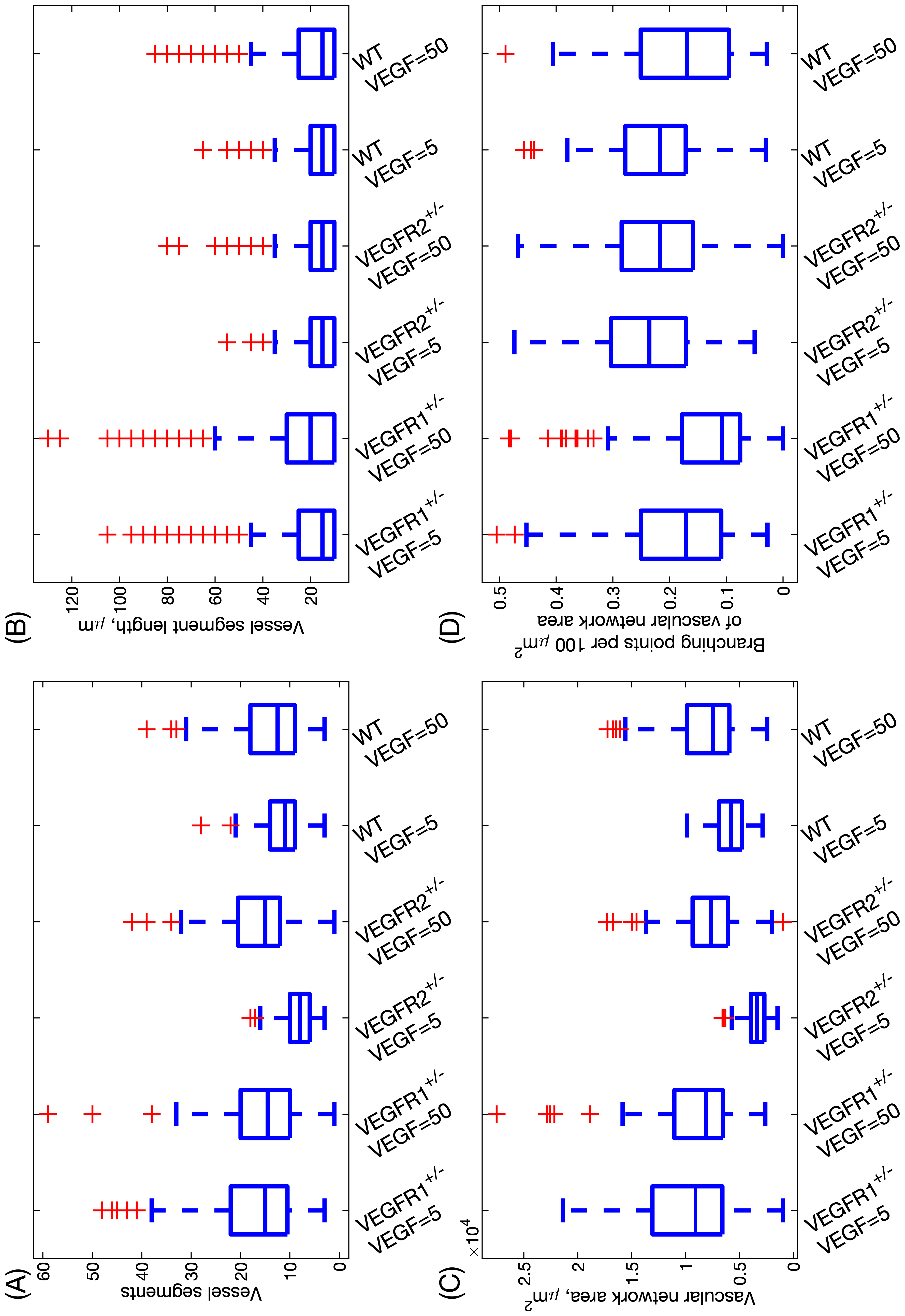

Supplement: S7 Fig — (A) Number of vessel segments. (B) Vessel segment length (μm). (C) Vascular network area (μm2) at the end of the numerical simulation. (D) Number of branching points per 100 μm2 of vascular network area. Details of definitions of these metrics can be found in S1 Text. In each boxplot, the central line indicates the median, and the horizontal edges of the box represent the 25th and 75th percentiles (for the bottom and top edges, respectively). The outliers are indicated by red cross symbols. Numerical simulation setup used is Setup 1 from S4 Table with final simulation time Tmax = 2.5. Parameter values are listed in S1 and S2 Tables for subcellular and cellular/tissue scales, respectively, except of those changed for mutant cells (see S1 Appendix). Results are averaged over 100 realisations for each experimental scenario. (TIF) [file pcbi.1008055.s008.tif]

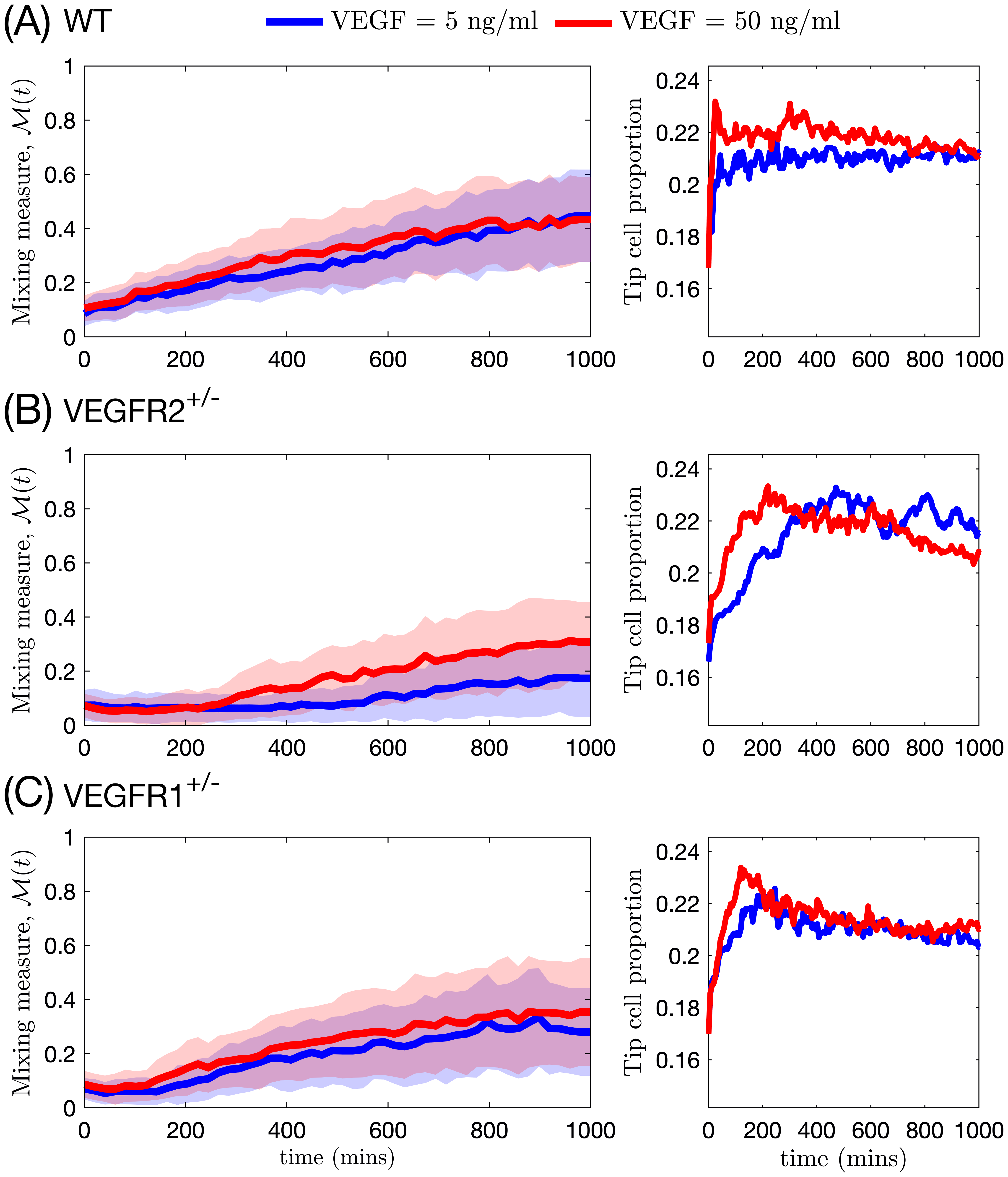

Supplement: S8 Fig — Left column plots show the mixing measure, M(t), as a function of time (the mean value is indicated by a thick line and standard deviation is shown by a band with corresponding colour). Right column plots demonstrate the evolution of tip cell proportion. Simulations were done for networks formed by (A) WT cells; (B) VEGFR2+/- mutant cells; and (C) VEGFR1+/- mutant cells. Numerical simulation setup used is Setup 1 from S4 Table with final simulation time Tmax = 2.5. Parameter values are listed in S1 and S2 Tables for subcellular and cellular/tissue scales, respectively, except of those changed for mutant cells (see S1 Appendix). Results are averaged over 100 realisations for each experimental scenario. (TIF) [file pcbi.1008055.s009.tif]

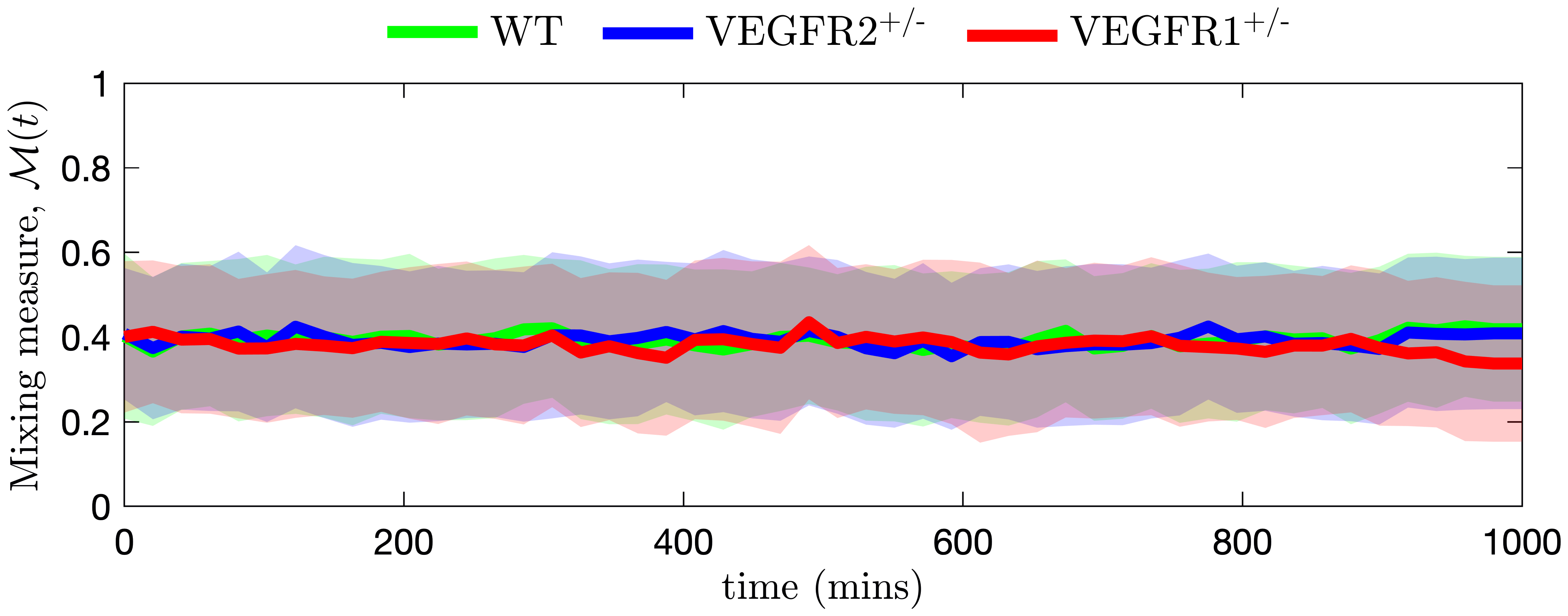

Supplement: S9 Fig — Plots of mixing measure, M(t), as a function of time for WT, VEGFR2+/- and VEGFR1+/- mutant cells for VEGF = 0 ng/ml (the mean value is indicated by a thick line and standard deviation is shown by a band of the corresponding colour). At this concentration of external VEGF, there is no effective sprout elongation, thus cells perform proteolysis-free random shuffling within already existing sprouts [52]. This leads to a steady state of the mixing measure for all cell lineages. Mean values are 0.39, 0.39 and 0.38 for WT, VEGFR2+/- and VEGFR1+/- cells, respectively. Numerical simulations were performed using Setup 1 from S4 Table and Tmax = 2.5. Parameter values are listed in S1 and S2 Tables for subcellular and cellular/tissue scales, respectively, except of those changed for mutant cells (see S1 Appendix). (TIF) [file pcbi.1008055.s010.tif]
