# Supplementary material for: A multiscale model of complex endothelial cell dynamics in early angiogenesis
Source: PLoS Comput Biol. 2021 Jan 7;17(1):e1008055. doi: 10.1371/journal.pcbi.1008055 (PMC7817011; doi:10.1371/journal.pcbi.1008055)
Supplement: S1 Table — Description and reference values used in simulations of the subcellular VEGF-Delta-Notch signalling. (PDF) [file pcbi.1008055.s011.pdf]

# A multiscale model of complex endothelial cell dynamics in early angiogenesis

Daria Stepanova[1,2 *], Helen M. Byrne[3], Philip K. Maini[3], Tomás Alarcón[4,1,2,5]

**1** Centre de Recerca Matemàtica, Bellaterra (Barcelona), Spain
**2** Departament de Matemàtiques, Universitat Autònoma de Barcelona, Bellaterra (Barcelona), Spain
**3** Wolfson Centre for Mathematical Biology, Mathematical Institute, University of Oxford, Oxford, UK
**4** Institució Catalana de Recerca i Estudis Avançats (ICREA), Barcelona, Spain
**5** Barcelona Graduate School of Mathematics (BGSMath), Barcelona, Spain

* dstepanova@crm.cat

| Parameter | Units | Description | Value used in simulations | Ref. |
|---|---|---|---|---|
| $R_s$ | $\mu m$ | Interaction radius. | 15 | estim., [1,2] |
| $b_N$ | $molec \cdot time^{-1}$ | Baseline Notch receptor expression. | 500 | [3,4] |
| $b_D$ | $molec \cdot time^{-1}$ | Baseline Delta ligand expression. | 800 | [3,4] |
| $b_{R2}$ | $molec \cdot time^{-1}$ | Baseline VEGFR2 expression. | 800 | [4] |
| $I_0$ | $molec$ | Activation threshold for NICD. | 100 | [4] |
| $R2^*_0$ | $molec$ | Activation threshold for activated VEGFR2. | 200 | [4] |
| $\lambda_{I,N}$ | $dimensionless$ | Weight factor characterising fold change of the production rate of Notch receptor depending on the NICD concentration. | 4.0 | [3,4] |
| $\lambda_{R2^*,D}$ | $dimensionless$ | Weight factor characterising fold change of the production rate of Delta ligand depending on the activated VEGFR2 concentration. | 2.0 | [4] |
| $\lambda_{I,R2}$ | $dimensionless$ | Weight factor characterising fold change of the production rate of VEGFR2 depending on the NICD concentration. | 0.0 | [4] |
| $n_N$ | $dimensionless$ | Cooperativity parameter for Hill function for NICD-dependent Notch up-regulation. | 2 | [5] |
| $n_D$ | $dimensionless$ | Cooperativity parameter for Hill function for activated VEGF-dependent Delta up-regulation. | 1 | [5] |
| $n_{R2}$ | $dimensionless$ | Cooperativity parameter for Hill function for NICD-dependent VEGFR2 repression. | 1 | [5] |
| $V$ | $molec$ | External VEGF. | 2500 (Fig 3E); $0 - 2500$ (Fig 3F); $\{0, 2500, 25000\}$ (in the rest of the simulations) | [3,4] |
| $D_{ext}$ | $molec$ | External Delta ligand. | $0 - 3000$ (Fig 3E and 3F); calculated from adjacent cells (in the rest of the simulations) | [3,4] |
| $N_{ext}$ | $molec$ | External Notch receptor. | 1000 (Fig 3E and 3F); calculated from adjacent cells (in the rest of the simulations) | [3,4] |
| $k_t$ | $molec^{-1} \cdot time^{-1}$ | Trans-binding rate for Notch receptor and Delta ligand. | $5.0e - 5$ | [5] |
| $k_c$ | $molec^{-1} \cdot time^{-1}$ | Cis-interaction rate for Notch receptor and Delta ligand. | $6.0e - 4$ | [5] |
| $k_v$ | $molec^{-1} \cdot time^{-1}$ | Binding rate for VEGFR2 and external VEGF. | $5.0e - 5$ | [4] |
| $\eta$ | $dimensionless$ | Endocytic regulation of Notch signalling. | 0.5 | estim., [6] |
| $\gamma$ | $time^{-1}$ | Degradation rate of proteins. | 0.1 | [4] |
| $\gamma_e$ | $time^{-1}$ | Degradation rate of activated receptors. | 0.5 | [4] |

**S1 Table. Baseline parameter values for the VEGF-Delta-Notch subcellular model.** Description and reference values used in simulations of the subcellular VEGF-Delta-Notch signalling.

# References


1. Du Y, Herath SC, Wang Qg, Wang Da, Asada HH, Chen PC. Three-dimensional characterization of mechanical interactions between endothelial cells and extracellular matrix during angiogenic sprouting. Scientific Reports. 2016;6:21362.

2. Ubezio B, Blanco RA, Geudens I, Stanchi F, Mathivet T, Jones ML, et al. Synchronization of endothelial Dll4-Notch dynamics switch blood vessels from branching to expansion. Elife. 2016;5:e12167.

3. Boareto M, Jolly MK, Lu M, Onuchic JN, Clementi C, Ben-Jacob E. Jagged–Delta asymmetry in Notch signaling can give rise to a Sender/Receiver hybrid phenotype. Proceedings of the National Academy of Sciences. 2015;112(5):E402–E409.

4. Boareto M, Jolly MK, Ben-Jacob E, Onuchic JN. Jagged mediates differences in normal and tumor angiogenesis by affecting tip-stalk fate decision. Proceedings of the National Academy of Sciences. 2015;112(29):E3836–E3844.

5. Sprinzak D, Lakhanpal A, LeBon L, Garcia-Ojalvo J, Elowitz MB. Mutual inactivation of Notch receptors and ligands facilitates developmental patterning. PLoS Computational Biology. 2011;7(6):e1002069.

6. Fortini ME, Bilder D. Endocytic regulation of Notch signaling. Current Opinion in Genetics & Development. 2009;19(4):323–328.
