# Supplementary material for: A multiscale model of complex endothelial cell dynamics in early angiogenesis
Source: PLoS Comput Biol. 2021 Jan 7;17(1):e1008055. doi: 10.1371/journal.pcbi.1008055 (PMC7817011; doi:10.1371/journal.pcbi.1008055)
Supplement: S2 Table — (PDF) [file pcbi.1008055.s012.pdf]

A multiscale model of complex endothelial cell dynamics in early angiogenesis

Daria Stepanova[1,2 *], Helen M. Byrne[3], Philip K. Maini[3], Tomás Alarcón[4,1,2,5]

1 Centre de Recerca Matemàtica, Bellaterra (Barcelona), Spain
2 Departament de Matemàtiques, Universitat Autònoma de Barcelona, Bellaterra (Barcelona), Spain
3 Wolfson Centre for Mathematical Biology, Mathematical Institute, University of Oxford, Oxford, UK
4 Institució Catalana de Recerca i Estudis Avançats (ICREA), Barcelona, Spain
5 Barcelona Graduate School of Mathematics (BGSMath), Barcelona, Spain

* dstepanova@crm.cat

**Parameter**	**Value**	**Parameter**	**Value**	**Parameter**	**Value**	**Parameter**	**Value**
R_c	$1.5h$	D_ω	1.0	c_{max}	1.0	Δ_l	0.01
E_{F1}	0.25	E_{F2}	0.7	s_{F1}	35.0	s_{F2}	10.0
p_{max}	0.26	s_p	0.0015	D_p	1500	a	7.0
n	2	K	20.0	k_m	2.6	k_D	0.0002
η_l	0.1	η_{max}	12.5	s_c	0.003	D_c	4200
γ_{max}	17.0	s_m	0.003	D_m	4200		

S2 Table. Parameter values of the cellular and tissue scales used in our simulations.
