# Supplementary material for: A multiscale model of complex endothelial cell dynamics in early angiogenesis
Source: PLoS Comput Biol. 2021 Jan 7;17(1):e1008055. doi: 10.1371/journal.pcbi.1008055 (PMC7817011; doi:10.1371/journal.pcbi.1008055)
Supplement: S3 Table — Here I is the set of all voxels; S is the set of all possible migration directions. DUnif[a, b] is a discrete uniform distribution over all integer numbers lying within the interval [a, b]; Unif[a, b] is the uniform distribution on the interval [a, b]. Baseline gene expression parameters for the VEGF-Delta-Notch signalling are listed in S1 Table. Δinit = 1.0 for all numerical simulations (this value, as, in general, for the value of the OL variable, is non-dimensional). The fluctuation parameter, ξ, is set to 0.1 in all numerical simulations. The exact values for cinit and minit are given for each numerical experiment in S4 Table, as well as the set of initial cell positions, Iinit. For the description of model variables see Table 1 in the main text. (PDF) [file pcbi.1008055.s013.pdf]

A multiscale model of complex endothelial cell dynamics in early angiogenesis

Daria Stepanova[1,2 *], Helen M. Byrne[3], Philip K. Maini[3], Tomás Alarcón[4,1,2,5]

1 Centre de Recerca Matemàtica, Bellaterra (Barcelona), Spain
2 Departament de Matemàtiques, Universitat Autònoma de Barcelona, Bellaterra (Barcelona), Spain
3 Wolfson Centre for Mathematical Biology, Mathematical Institute, University of Oxford, Oxford, UK
4 Institució Catalana de Recerca i Estudis Avançats (ICREA), Barcelona, Spain
5 Barcelona Graduate School of Mathematics (BGSMath), Barcelona, Spain

* dstepanova@crm.cat

**Variable**	**Indexes**	**Description**
$E_i = 1$<br>$E_i = 0$	$i \in \mathcal{I}_{init}$<br>$i \in \mathcal{I} \setminus \mathcal{I}_{init}$	Initial distribution of cell nuclei.
$N_i = \text{Unif}[(1-\xi)b_N, (1+\xi)b_N]$<br>$D_i = \text{DUnif}[(1-\xi)b_D, (1+\xi)b_D]$<br>$I_i = \text{DUnif}[(1-\xi)I_0, (1+\xi)I_0]$<br>$R2_i = \text{DUnif}[(1-\xi)b_{R2}, (1+\xi)b_{R2}]$<br>$R2_i^* = \text{DUnif}[(1-\xi)R2_0^*, (1+\xi)R2_0^*]$<br>$N_i = D_i = I_i = R2_i = R2_i^* = 0$	$i \in \mathcal{I}_{init}$<br>$i \in \mathcal{I} \setminus \mathcal{I}_{init}$	Cells are initialised with ligand/receptor numbers corresponding to their baseline gene expression with a correction for random fluctuations included via the parameter ξ. At the voxels where there is no cell nucleus, subcellular variables are initialised with the value zero.
$l_i^{s_{init}} = 2\Delta_{init}$	$i \in \mathcal{I}_{init}$	The alignment of ECM fibrils for voxels where cells were initially placed in the direction $s_{init} \in \mathcal{S}$.
$l_i^s = \text{Unif}[0, \Delta_{init}]$<br>$l_i^s = \text{Unif}[0, \Delta_{init}]$	$i \in \mathcal{I} \setminus \mathcal{I}_{init}$, $\forall s \in \mathcal{S}$<br>$i \in \mathcal{I}_{init}$, $\forall s \neq s_{init} \in \mathcal{S}$	The alignment of ECM fibrils for the rest of the voxels is initialised with a small random value in a given range, $[0, \Delta_{init}]$, imitating random orientation of fibrils prior to their realignment due to cell migration.
$c_i = c_{init}$<br>$c_i = c_{max}$	$i \in \mathcal{I}_{init}$<br>$i \in \mathcal{I} \setminus \mathcal{I}_{init}$	The ECM concentration at the voxels with cells is equal to $c_{init} \in [0, c_{max}]$ (specified for each numerical experiment). For other voxels, the ECM is assumed to be unchanged, thus equal to the maximum ECM concentration, c_{max}.
$m_i = m_{init}$<br>$m_i = 0$	$i \in \mathcal{I}_{init}$<br>$i \in \mathcal{I} \setminus \mathcal{I}_{init}$	The concentration of BM components at the voxels with cells is equal to $m_{init} \in [0, 1]$ (specified for each numerical experiment). For other voxels, no BM components have been deposited, thus the concentration is set to zero.

S3 Table. Initial conditions for numerical simulations. Here $\mathcal{I}$ is the set of all voxels; $\mathcal{S}$ is the set of all possible migration directions. DUnif$[a, b]$ is a discrete uniform distribution over all integer numbers lying within the interval $[a, b]$; Unif$[a, b]$ is the uniform distribution on the interval $[a, b]$. Baseline gene expression parameters for the VEGF-Delta-Notch signalling are listed in S1 Table. $\Delta_{init} = 1.0$ for all numerical simulations (this value, as, in general, for the value of the OL variable, is non-dimensional). The fluctuation parameter, ξ, is set to 0.1 in all numerical simulations. The exact values for c_{init} and m_{init} are given for each numerical experiment in S4 Table, as well as the set of initial cell positions, $\mathcal{I}_{init}$. For the description of model variables see Table 1 in the main text.
