# Supplementary material for: A multiscale model of complex endothelial cell dynamics in early angiogenesis
Source: PLoS Comput Biol. 2021 Jan 7;17(1):e1008055. doi: 10.1371/journal.pcbi.1008055 (PMC7817011; doi:10.1371/journal.pcbi.1008055)
Supplement: S4 Table — For each setup of numerical simulation we specify the lattice dimensions, NIx and NIy; the set of indices corresponding to the vascular plexus, IVP; the initial cell nuclei positions, Iinit; the initial polarisation direction, sinit; the initial ECM and BM concentrations, cinit and minit, respectively; the VEGF distribution over the lattice, V; and cell line used in simulations. (PDF) [file pcbi.1008055.s014.pdf]

A multiscale model of complex endothelial cell dynamics in early angiogenesis

Daria Stepanova[1,2 *], Helen M. Byrne[3], Philip K. Maini[3], Tomás Alarcón[4,1,2,5]

1 Centre de Recerca Matemàtica, Bellaterra (Barcelona), Spain
2 Departament de Matemàtiques, Universitat Autònoma de Barcelona, Bellaterra (Barcelona), Spain
3 Wolfson Centre for Mathematical Biology, Mathematical Institute, University of Oxford, Oxford, UK
4 Institució Catalana de Recerca i Estudis Avançats (ICREA), Barcelona, Spain
5 Barcelona Graduate School of Mathematics (BGSMath), Barcelona, Spain

* dstepanova@crm.cat

Set-up	Specifications	Figures
**Setup 1:** Individual sprout	$N_I^x = 25,\ N_I^y = 29$<br>$\mathcal{I}_{init} = \{i = (i_x, i_y)^T :\ i_x = 1, 2,\ i_y = 14, 15\}$<br>$\mathcal{I}_{VP} = \{i = (1, i_y)^T :\ i_y = 14, 15\}$<br>$s_{init} = r$<br>$c_{init} = 0.0$, $m_{init} = 1.0$<br>$V \in \{0, 2500, 25000\}$, uniform distribution over the lattice, which correspond to 0, 5 and 50 ng/ml VEGF concentration, respectively. The exact value specified in the text.<br>Simulations with this setup are performed with WT and mutant cells (see S1 Table and S1 Appendix, respectively).	For WT cells: Figs 7, 8, 10, 13, 14 and S7-S9 Figs. For mutant cells: Fig 15, S3-S9 Figs.
**Setup 2:** Vessel in VEGF gradient	$N_I^x = 25,\ N_I^y = 29$<br>$\mathcal{I}_{init} = \{i = (i_x, i_y)^T :\ i_x = 12, 13,\ i_y = 1, \ldots, 29\}$<br>$\mathcal{I}_{VP} = \mathcal{I}_{init}$<br>$s_{init}$ - not specified<br>$c_{init} = c_{max}$, $m_{init} = 0.0$<br>$V(i_x, i_y) = 2500 \frac{i_y}{N_I^y}$. This corresponds to a VEGF gradient linearly increasing from 0 to 5 ng/ml along the y-axis.<br>Simulations with this setup are performed with WT cells (see S1 Table).	Fig 9
**Setup 3:** Cell bead	$N_I^x = 25,\ N_I^y = 29$<br>$\mathcal{I}_{init} = \{i = (i_x, i_y)^T :\ (i_x - 13)^2 + (i_y - 9)^2 \leq 5,\ i \in \mathcal{I}\}$<br>$\mathcal{I}_{VP} = \mathcal{I}_{init}$<br>$s_{init}$ - not specified<br>$c_{init} = c_{max}$, $m_{init} = 0.0$<br>$V(i_x, i_y) = 2500 \frac{i_y}{N_I^y}$. This corresponds to a VEGF gradient linearly increasing from 0 to 5 ng/ml along the y-axis.<br>Simulations with this setup are performed with WT cells (see S1 Table).	Fig 11
**Setup 4:** Linear sprout	$N_I^x = 25,\ N_I^y = 2$<br>$\mathcal{I}_{init} = \{i = (i_x, i_y)^T :\ i_x = 1, \ldots, 25,\ i_y = 1, 2\}$<br>$\mathcal{I}_{VP} = \mathcal{I}_{init}$<br>$s_{init} = r$<br>$c_{init} = 0.0$, $m_{init} = 1.0$<br>$V = 15000$, corresponding to a constant uniform distribution of VEGF at the concentration 30 ng/ml (used in [1]).<br>Simulations with this setup are performed with mutant cells (S1 Appendix).	Fig 12, Table 3

S4 Table. Setups of simulation experiments. For each setup of numerical simulation we specify the lattice dimensions, N_I^x and N_I^y; the set of indices corresponding to the vascular plexus, $\mathcal{I}_{VP}$; the initial cell nuclei positions, $\mathcal{I}_{init}$; the initial polarisation direction, s_{init}; the initial ECM and BM concentrations, c_{init} and m_{init}, respectively; the VEGF distribution over the lattice, V; and cell line used in simulations.

References

1. Jakobsson L, Franco CA, Bentley K, Collins RT, Ponsioen B, Aspalter IM, et al. Endothelial cells dynamically compete for the tip cell position during angiogenic sprouting. Nature Cell Biology. 2010;12(10):943.
