# Supplementary material for: A multiscale model of complex endothelial cell dynamics in early angiogenesis
Source: PLoS Comput Biol. 2021 Jan 7;17(1):e1008055. doi: 10.1371/journal.pcbi.1008055 (PMC7817011; doi:10.1371/journal.pcbi.1008055)
Supplement: Attachment — Submitted filename: Response to reviewers.pdf [file pcbi.1008055.s020.pdf]

We thank the reviewers for their positive and constructive comments on our manuscript. We have now revised it in line with their suggestions and include a point-by-point response to these suggestions. We hope you will agree that the revised manuscript is now suitable for publication in *PLOS Computational Biology*.

This rebuttal letter is organised as follows:

- Throughout the letter, we use the following abbreviations:

EC	endothelial cell;
VEGF	vascular endothelial growth factor;
ECM	extracellular matrix;
BM	basement membrane.

- Numbering of equations, figures, tables and lines refers to the main text of the revised manuscript unless otherwise specified.
- For reviewers' convenience, we included in this letter excerpts of the revised manuscript showing the corrections related to their comments (highlighted in blue colour).
- Bibliography is listed in the end of this document.

Reviewer 1

1. In line 180 it is mentioned that "...actin filaments close to the membrane... can bind more VEGF and ECM proteins." However, actin filaments do not bind VEGF and ECM proteins directly.

We agree that this sentence was not clear and have now revised it. See correction on lines 177-186 of the revised manuscript:

VEGF-induced actin polymerisation and focal adhesion assembly result in substantial cytoskeletal remodelling as required for cell migration [Rousseau et al., 2000]. This includes actin remodelling to form filopodia and lamellipodia, stress fibre formation, and focal adhesion turnover [Lamalice et al., 2007]. A crucial consequence of such remodelling is that ECs acquire front-to-rear polarity which coincides with the direction of their migration [Watanabe et al., 2005]. Membrane protrusions, such as filopodia and lamellipodia, increase the cell surface area at the leading edge. More VEGF receptors and integrins, which bind to ECM components, become activated at the cell front to further reinforce the cytoskeleton remodelling and polarisation [Reinhart-King et al., 2005]. This mechanism is known as taxis (e.g. chemotaxis, haptotaxis) and allows ECs to sense extracellular cues and migrate towards them.

2. In lines 188/189 and then on the results section (lines 569 onwards) the authors mention the "brush-border" effect. The authors should compare directly the model results with experiments on how branch density increases with VEGF concentration. In particular experiments of our collaborators indicate that there is a saturation VEGF concentration above which the number of sprouts starts decreasing. Is the model able to reproduce this VEGF concentration

saturation? Here, if I understood correctly, the proposed mechanism is: more VEGF leads to more tip cells and Delta expression, which lead to more ECM degradation and more branches. So the question is what is the behaviour of the simulated Notch-Delta stochastic model and ECM proteolysis at high VEGDF concentrations.

In the model proposed in this paper, the ECs' response to VEGF signalling saturates. In particular, the ECM proteolysis and BM assembly rates, $\eta_c(D_i)$ and $\gamma_m(D_i)$, respectively, are sigmoid functions of subcellular Delta levels, D_i (see Eqs (19), (21)). Thus, an increase in VEGF signalling above a certain threshold and, as a consequence, an increase in Delta levels, will not result in uncontrolled matrix degradation and vessel branching; it will saturate. This is now clarified in lines 515-518 and 524-527 of the revised manuscript:

Here, in order to account for the natural saturation in EC proteolytic ability, we assume a sigmoidal functional form for $\eta_c(D_i)$ with positive parameters D_c and s_c (which correspond to the threshold level of Delta for initiation of ECM proteolysis and sharpness of EC response, respectively) and maximum value η_{max}.
...
where, again, a sigmoidal functional form is assumed for $\gamma_m(D_i)$ with positive parameters D_m and s_m (which correspond to the threshold level of Delta for initiation of BM assembly and sharpness of EC response, respectively) and maximum value γ_{max}.

3. The Notch dynamics is modelled though a master equation simulated via a NSV algorithm. Can the authors refer to experimental evidence of stochastic Notch-Dll4 phenotype switching? Bentley et al have done extensive experiments on the time dependence of Delta-4 expression in endothelial cells and find that the oscillations in expression are periodic and can be explained by the coordinated response of groups of endothelial cells to high levels of VEGF. How do the results of the simulations depend on the stochastic nature of Notch-Delta dynamics? Also, how does the presence of ECs intermediate states (neither stalk not tip cell, predicted in Bentley et al models) would affect the model predictions for EC mixing?

Although large numbers of proteins (as in the case of the subcellular model of VEGF-Delta-Notch signalling used in this work) reduce the noise in the system, the fact that ECs contain finite numbers of proteins implies that some level of stochasticity is always present. Moreover, subcellular signalling, i.e. cell phenotype, is closely related to branching, ECM remodelling and cell rearrangements. Thus, it is natural to explore the role of noise in the Delta-Notch system during the early stages of angiogenesis. Particularly, since stochasticity allows transitions between stable steady states (phenotype switches) to occur in regions of bistability, such transitions cannot be achieved in a deterministic model.

This has been clarified in lines 266-275 of the revised manuscript:

The **subcellular scale** defines the gene expression pattern of individual cells. Since ECs contain a finite number of proteins, some level of noise is always present in the system. Thus, we implement a stochastic model of the VEGF-Delta-Notch signalling pathway to describe the temporal dynamics of the number of ligands/receptors for each cell. This pathway is known to produce bistable behaviour. Cells exhibit either high Delta and VEGFR2 and low Notch levels (the tip phenotype) or low Delta and VEGFR2 and high Notch levels (the stalk phenotype). Stochasticity allows random transitions between these phenotypes in regions of bistability, behaviour which cannot be achieved in a deterministic model.

Regarding experimental evidence for stochastic Delta-Notch phenotype switching, it is widely accepted that cell phenotype (tip cell, stalk cell) is not a final fate but is dynamically updated [Arima et al., 2011,

Jakobsson et al., 2010, Bentley et al., 2009, Bentley et al., 2014]. In the works of Bentley et al., detailed models for cell cross-talk are developed and extensive analysis performed for small cell numbers. The main focus of our paper is to connect subcellular cell signalling with cell migration and vascular network formation. Thus, the subcellular model we employ is as detailed as necessary in order to admit phenotype switching. At the same time, the model is less complex than the work of Bentley et al. so that it is feasible to run simulations involving hundreds of cells in a network. An extension of the subcellular model taking into account predictions by Bentley et al. regarding intermediate (hybrid) EC phenotype and oscillatory cell phenotype synchronisation in high levels of VEGF will be the subject of future work.

4. ECs are polarised and this polarisation determines EC movement in the model. However, cell contacts are not influenced by the polarisation direction, and are determined instead by the other cells within a circular neighbourhood. Have the authors explored the possibility of an elliptical neighbourhood aligned with the polarisation direction to determine Notch-Delta interactions? How would this alteration affect the model results?

The possibility of an elliptical neighbourhood aligned with the cell polarity has not been explored in this work. Given the characteristic branching pattern of a vascular network, a cell's neighbourhood is a segment of a sprout (see Fig 4). Thus, the lateral regions of a circular neighbourhood, which would not be taken into account by an elliptical neighbourhood, are typically empty. A change from a circular to an elliptic neighbourhood would only require an adjustment of the threshold parameters, E_{F1} and E_{F2}, in the neighbourhood function, $F(E^N)$ (see Eq (9)).

We have now clarified this in the caption of Fig 4 of the revised manuscript:

In this work, we assume a circular neighbourhood for cell-cell interactions within the so-called *interaction radius*, R_c, for the cellular scale (here it is drawn to have the same value as in our simulations, $R_c = 1.5h$, where h is the voxel width). This allows cells to interact beyond their immediate neighbours as, for example, the cell with the nucleus marked by a red star and the focal green cell. Other geometries (e.g. elliptical neighbourhood aligned with cell polarity vector) are unlikely to alter the model behaviour significantly since, in sprouting structures, lateral regions of the circular neighbourhood, which would be ignored by an elliptic neighbourhood, are typically empty.

5. Explain in greater detail the reasoning behind the terms in equation (9).

The form of the neighbourhood function, $F(E^N)$ (Eq (9)), was chosen in order to phenomenologically capture certain features of cell-to-cell interactions. It is composed of an increasing sigmoid (the first term in Eq (9)) positioned at E_{F1} with slope s_{F1} and a decreasing sigmoid (the second term in Eq (9)) positioned at E_{F2} with slope s_{F2}. Taken together, and with suitable scaling such that $F(E^N) \geqslant 0$, with these components, the neighbourhood function grows as E^N increases from small values, attains a maximum for $E_{F1} < E^N < E_{F2}$ and decreases for large values of E^N (mimicking the inhibiting effect of crowding on cell movement) (see Fig 5(E)). In biological experiments [Sugihara et al., 2015], it was shown that when a leader cell loses contacts with its neighbours (laser ablation experiments) it halts until the following cells reach it. This effect is captured by the increasing part of $F(E^N)$: when the number of neighbours around a cell is below the sigmoid threshold E_{F1} the probability of cell movement decreases rapidly to 0, thus the migration transition (Eq (7)) goes to 0 as well. Similarly, when there are many ECs in cell's neighbourhood, its migration slows down. This is reflected in the decreasing part of $F(E^N)$ when above the threshold E_{F2} in the number of neighbouring cells, cell migration is slowed down and eventually stopped in the high density regions.

This has been clarified in lines 422-435 of the revised manuscript:

The effect of cell-cell adhesion on cell migration is incorporated via the so-called *neighbourhood function*, $F(E_i^N)$. Its argument, E_i^N, represents the number of ECs in a cell's local neighbourhood (red-coloured cells in Fig 4). The functional form of the neighbourhood function was chosen in order to phenomenologically capture the way in which EC behaviour depends on the cell-cell contacts (see Fig 5(E)). In biological experiments, it was shown that when a cell loses contact with its neighbours (laser ablation experiments in [Sugihara et al., 2015]) it halts until the following cells reach it. This is captured by the increasing part of $F(E_i^N)$; when the number of neighbours around a cell is below the first threshold, E_{F1}, the probability of cell movement decreases rapidly to zero, thus the migration transition (Eq (7)) goes to 0 as well. Similarly, when there are many ECs in a cell's neighbourhood, its movement slows down. This is accounted for by the decreasing part of $F(E_i^N)$ when the number of neighbouring cells exceeds the second threshold value, E_{F2}, cell movement is slowed down and eventually halts in regions of high cell density.

6. The branching angles in the model are strongly influenced by the lattice. Once the new tip cell starts exploring other directions, its movement is locked at a 60 degree angle relative to the initial vessel since it has no other option. If there were no lattice I would expect that the new tip cell could in principle bifurcate with a wide range of possible angles relative to the initial vessel. In particular the new vessel could grow almost parallel to the original vessel; I would even expect that angles close to zero would be the most probable. How can the model become lattice-free and achieve more realistic vessel geometries?

In the model, individual cell polarity angle is sampled from the von Mises distribution, with the mean defined by the sprout elongation direction and its variance by the function $\kappa(D, m)$ which depends on Delta levels, D, (cell phenotype) and the concentration of deposited BM components, m. It can be seen from Fig 5(D) that for the values of $\kappa(D, m)$ considered in our paper, the von Mises distribution is concentrated around its mean value (see Fig 5(C)). Thus, the angles sampled from this distribution are typically close to the mean value, as the reviewer notes. In this case, ECs follow the direction of sprout elongation, that is also the most probable scenario from the biological point of view. Less frequently, when a sampled angle lies outside the direction of sprout elongation, the cell migrates in that new direction and a new branch is formed. As experimental evidence for this modelling assumption, we note that in [Arima et al., 2011], a coordination statistic was calculated which quantifies the angle of cell migration relative to the direction of sprout elongation. As expected, the distribution of the coordination angle is centred at 0 with variance decreasing as VEGF concentration increases. Thus, the generalisation of the model to an off-lattice one is straightforward but more computationally intensive. This is why we use a lattice-based model. Unfortunately, using the current framework we cannot distinguish vessels that grow almost parallel since only the positions of cell nuclei are tracked.

7. The model has a very large number of parameters. The authors calibrate the model choosing a combination of parameters by comparison to experiments. I expect that there are other possible choices of parameters that will lead to a similar performance of the model. In which order the authors chose the parameters? Which ones were set to fixed values and which ones were fit by the experimental results?

The subcellular scale model used in this work is based on the lateral inhibition model of the Delta-Notch signalling pathway developed in [Boareto et al., 2015b, Boareto et al., 2015a], with VEGF-induced cell activation as in [Sprinzak et al., 2010]. An extensive analysis of the models, including calibration and validation, was carried out in those references and formed the basis for our parameter estimation for the subcellular scale. Thus, all the parameters of this scale (see S1 Table) were fixed. By contrast, the cellular

and tissue scale parts of the multiscale model are novel. Parameters corresponding to these scales (see S2 Table) were calibrated and then validated using experimental data. In addition, we performed an extensive sensitivity analysis for the parameters corresponding to the cellular and tissue scales. The results can be found in **Results** section of the main text (subsection **Sensitivity analysis**, lines 700-748 of the revised manuscript) and Supplementary material, S1 Text.

Sensitivity analysis

To ascertain how variation in the baseline parameter values of our model affects the behaviour of the system, we have performed an extensive sensitivity analysis. Since the subcellular VEGF-Delta-Notch model has already been calibrated and validated independently [Boareto et al., 2015a], we have focused our analysis on the cellular and tissue scale parameters (see S2 Table). Briefly, we have performed our analysis by fixing all the parameters except one at their baseline values, and then vary the focal parameter by $\pm 0.1\%$, $\pm 5\%$, $\pm 10\%$, $\pm 15\%$, and $\pm 20\%$. This procedure is repeated for each of the tissue and cellular scale parameters. In order to quantify the impact of the variation of each parameter on both EC behaviour and network structure we have measured the following quantities:

- anterograde cell proportion (directionality metric);
- orientation;
- displacements;
- number of branching points per 100 μm^2 of vascular network area;
- number of vessel segments;
- vessel segment lengths.

Each of these metrics has been measured over 100 realisations of the multiscale system. For a full account of the details, see Supplementary material, S1 Text.

The results of our sensitivity analysis are summarised in Fig 13 and S2 Fig. Our analysis shows that system behaviour is robust to variations in most of the model parameters considered. This is indicated by the central cluster in Fig 13 and S2 Fig, highlighted in magenta, which represents those scenarios that exhibit very small deviations from the baseline behaviour. By contrast, variations of a small number of parameters produce significant deviation from the baseline behaviour (see Table 4, Fig 13 and S2 Fig).

Specifically, we observe that an increase in D_m and a decrease in both D_c and K induce excessive branching, with shorter average vessel length (see Fig 13, hyper-branching region highlighted in brown). By contrast, a decrease in D_m and an increase in both D_c and K induce less branched networks, with longer average vessel length (see Fig 13, hypo-branching region highlighted in grey). These results are in agreement with well-known features of tumour vasculature, where the tumour microenvironment inhibits vessel stabilisation; specifically, it hinders the formation of the basal membrane in tumour vasculature, yielding aberrant, excessively branched, networks [Papetti and Herman, 2002]. This phenomenon can be realised by increasing D_m. Furthermore, proteolysis is also up-regulated during tumour-induced angiogenesis due to secretion of MMPs by cancer cells. Proteolysis reduces the resistance experienced by the ECs as they migrate towards the tumour [Papetti and Herman, 2002]. This effect can be accounted for phenomenologically by a reduction in D_c. Increased stimulation with growth factors that can also bind to VEGF receptors (as in pathological angiogenesis [Gupta and Qin, 2003, Papetti and Herman, 2002]) can reduce the response of the ECs to chemotactic stimuli, due to high occupancy of receptors all over the cell membrane, thus shifting cell behaviour to chemokinesis (non-directional cell migration) [Plank and Sleeman, 2003, Barkefors et al., 2008]. In our model, this transition is controlled by the cell exploratoriness, κ (see Eq (9)): for high values of κ (i.e. higher values of K) cell migration is directed along the sprout elongation vector, whereas for small values of κ (i.e. smaller values of K) cells exhibit exploratory behaviour corresponding to chemokinesis.

Par.	Description	Ref. equation	Metrics affected	Effect
D_c	Threshold of level of Delta for initiation of ECM proteolysis	Eq (19)	All	Hyper-branching: D_c ↓; hypo-branching: D_c ↑
D_m	Threshold of level of Delta for initiation of BM assembly	Eq (21)	All	Hypo-branching: D_m ↓; hyper-branching: D_m ↑
K	Cell exploratoriness, i.e. controls the variance of the von Mises distribution	Eq (14)	All	Hyper-branching: K ↓; hypo-branching: K ↑
E_{F1}	Threshold of the level of cell-cell contact necessary for cell movement initiation	Eq (9)	Orientation, directionality	Hyper-branching: E_{F1} ↓
E_{F2}	Threshold of the inhibitory effect of crowding on cell movement	Eq (9)	Orientation, directionality	Hypo-branching: E_{F2} ↓

Table 4. **Sensitivity analysis: parameters producing significant deviation in system behaviour from the baseline scenario.** ↓ stands for decrease of the focal parameter, ↑ - increase of the focal parameter.

Our model thus predicts that changes in D_c, D_m and K are likely to occur in tumour-induced angiogenesis. This prediction is supported by current knowledge regarding the effects of the presence of a tumour in the microenvironment [Gupta and Qin, 2003, Papetti and Herman, 2002, Plank and Sleeman, 2003].

8. In page 28 the authors show that the ratio of cells undergoing anterograde movement relative to retrograde movement increases with VEGF levels. The authors should better describe the mechanisms underlying this finding. How does the probability for the cell to undergo retrograde movement depends on the microenvironment (ECM concentration and alignment, basement membrane, VEGF...) and on the relative position and phenotype of neighbouring cells?

The directionality statistic was computed for comparison with experimental results from [Arima et al., 2011], where it was claimed that VEGF enhances EC migration in the direction of sprout elongation (i.e. the ratio of anterograde to retrograde cells increases with increasing VEGF concentration). However, no explanation of the phenomenon was provided. In our model, at low VEGF concentrations, cell proteolysis is slow and sprout elongation is limited. Thus, cell movement is mostly constrained to cell rearrangements within existing sprouts, i.e. if one cell moves forward, it overtakes another cell that has to move backwards. This is why at VEGF = 0 ng/ml, anterograde and retrograde movements balance. VEGF-induced up-regulation of Delta enables ECs to degrade the surrounding matrix at a faster rate (see Eqs. (18)-(19)). This increases the probability that ECs at the leading edge of a sprout move forward and, hence, increases the ratio of anterograde to retrograde movement.

This has been clarified in the revised manuscript (see lines 627-636):

Concerning the directionality statistic (Fig 10(D)-(F)), we note that when VEGF=0 ng/ml the numbers of anterograde and retrograde cells in the experiments [Arima et al., 2011] (Fig 10(F)) and numerical simulations (Fig 10(E)) are approximately equal. In this scenario, ECM proteolysis is slow and cell migration is mostly constrained to existing sprouts. Thus, any anterograde movement is an overtaking event in which the overtaken cell has to perform a retrograde displacement. As the VEGF concentration increases, ECM proteolysis (see Eqs (18)-(19)) increases and more cells at the leading edge of sprouts can invade the surrounding ECM, elongating the sprouts. This leads to an increase in the ratio of anterograde to retrograde

moving cells with the VEGF concentration.

9. In line 741 the authors mention that "The vascular networks generated by our model are in good agreement with experimental observations". Well, not quite: there are several features that are in good agreement but the general network aspect is quite different from experimental results (notably vessel structure depends dramatically on the conditions in which vessels grow, 2D vs 3D, retina vs heart, ex-vivo vs in-vivo, etc..). I suggest the authors to soften the sentence.

The model was calibrated and validated using experimental data from in vitro experiments of sprouting angiogenesis at early stages ($<$ 24 hours). As noted by the reviewer, branching structures are diverse and it is difficult to reproduce all of the trends. The merit of our model is that it can reproduce the general trends of angiogenic sprouting such as chemotactic sensitivity, cell rearrangements and branching upon movement, although branching patterns vary depending on the experimental setup. The sentence mentioned by the reviewer has been revised as suggested; see lines 820-824 of the revised manuscript:

The vascular networks generated by our model are capable of reproducing the general traits of sprouting angiogenesis: the networks exhibit branching patterns (see Figs 7-8), sprout elongation is enhanced in higher VEGF stimulation (see Fig 8) and the brush-border effect can be observed in networks grown in VEGF gradients (see Fig 9).

Reviewer 2

1. The authors have incorporated the aspect of cis-inhibition in their model, but no robust experimental evidence exists for cis-inhibition in angiogenesis. How do the results presented change or are expected to change if cis-inhibition is weakened or removed? The role of cis-inhibition in Notch-Delta signalling has been well-explored in terms of cell-fate decisions (Sprinzak et al. Nature 2010, Jolly et al. New J Physics 2015).

The subcellular model used in this work is based on the lateral inhibition model of the Delta-Notch signalling pathway with VEGF-induced cell activation presented in [Boareto et al., 2015b, Boareto et al., 2015a, Sprinzak et al., 2010]. Cis-interaction between a Notch receptor and a Delta ligand is taken into account in these models. In more detail, cis-interaction is shown to increase the stability of the steady states (cell phenotypes); but the number of steady states does not change when the intensity of cis-interaction varies [Boareto et al., 2015a] (see Figure S1 in [Boareto et al., 2015a]). In addition, the effect of variations in the intensity of Delta-Notch cis-inhibition has been extensively investigated in several modelling papers on angiogenesis [Boareto et al., 2015a, Koon et al., 2018, Boas and Merks, 2015, Vega et al., 2020]. Therefore, in this paper we focussed on the relationship between cell phenotype, cell migration and vascular network structure rather than performing a detailed exploration of the intracellular signalling of ECs. Finally we note that in Supplementary material, S1 Text, SM-Fig 5, we illustrate how phenotype patterning changes in a cell monolayer as cis-inhibition parameter varies. We have revised the manuscript and included a reference to these results in lines 388-393 of the revised manuscript:

We also investigated how phenotype patterning changes within a monolayer as cis-inhibition intensity varies (see Supplementary material, S1 Text). For low values of the cis-inhibition parameter, κ_c, typical chessboard tip-stalk pattern is produced; as κ_c increases, ECs with tip phenotype can become adjacent to each other, thus increasing the time to patterning, since the lateral inhibition is weakened (see SM-Fig 5 in

S1 Text).

2. The authors discuss about cell mixing/rearrangements in sprouting angiogenesis. Does this mixing continue in homeostatic conditions? If yes, what is the need and implication of such mixing? If not, what mechanisms lead to stopping this behaviour? Is this mixing a completely cell-autonomous decision or also influenced by biochemical and/or biomechanical crosstalk with the neighbours? Recent evidence about cell rearrangements has been reported in collective cell migration (Zhang et al. PNAS 2019). The authors should discuss similarities and differences with the mechanisms at play there.

In angiogenesis, homeostatic conditions (i.e. cell quiescence and sprout maturation) are achieved via EC interactions with smooth muscle cells (pericytes and murine cells), isolation of ECs from the ECM by a newly deposited BM and a blood flow-induced switch from front-to-rear to basal-ventral polarity of ECs [Potente and Carmeliet, 2017]. At this stage, cell migration ceases and, consequently, there are no cell rearrangements. In this paper, we focus on the early stages of angiogenic sprouting (i.e. before the homeostasis is reached) consequently, discussions of whether cell mixing occurs under homeostasis are not relevant for the current work.

Regarding the second reviewer's question of whether cell mixing is a cell-autonomous phenomenon, our results suggest that the phenomenon of cell rearrangements originates from cell crosstalk. In particular, factors that influence cell movement are the cell overtaking probability and the rates of ECM remodelling which are all phenotype dependent. Cell phenotype is established at the subcellular scale via biochemical contact-dependent crosstalk between ECs. We clarified this point on lines 784-787 of the revised manuscript:

This supports our hypothesis that cell shuffling is directly related to the phenotypic specifications of ECs. Thus, we note that the cell rearrangement phenomenon is not a cell-autonomous decision but rather a result of contact-dependent EC crosstalk, which leads to EC phenotype specification.

Collective cell migration is characteristic not only of endothelial cells during angiogenesis. Cancer cells [Zhang et al., 2019], as indicated by the reviewer, as well as neural crest cells [Szabó and Mayor, 2016, Giniūnaitė et al., 2020], epithelial and mesenchymal cells [Theveneau and Mayor, 2013] and other cell types are also capable of collective migration and ECM invasion. Although these processes share many similarities (e.g. cell-cell and cell-matrix adhesion, mechanisms of cell locomotion), each phenomenon has its own characteristic features. For example, unlike cancer cells, ECs form branching vascular networks. Further, the ways in which they interact with other cells (e.g. smooth muscle cells) and remodel the ECM differ from those used by cancer cells. Since both ECs and cancer cells are glycolytic, they have similar metabolic regulation which is essential or cell migration. However, to our knowledge, the relationship between cell metabolism and cell rearrangements in angiogenesis has not yet been investigated. In the absence of experimental data or hypotheses on which to base such model extensions, we postpone consideration of these effects to future work.

3. The authors have implemented a 2D framework of Notch signaling coupled with VEGF signalling. They should briefly discuss how similar or different their frameworks is from other attempts to simulate Notch signaling in 2D and its coupling with cell-fate decisions such as EMT (Boareto et al. J R Soc Interface 2016).

The subcellular model of VEGF-Delta-Notch signalling incorporated in our model is based on the works of [Boareto et al., 2015b, Boareto et al., 2015a, Sprinzak et al., 2010]. A distinguishing feature of our implementation is that cell-cell interactions are allowed to be non-local, within a circular neighbourhood of the

subcellular interaction radius, R_s. This is because we do not account explicitly for cell shape and assume that cell position is specified by the position of its nucleus. We investigated the influence of varying the interaction radius, R_s, on steady state phenotype patterning in S1 Fig. In addition, we would like to highlight that the main goal of this paper is not to investigate in great detail the subcellular signalling pathways of ECs but rather to couple cell phenotype specification to cell migration and interactions with the extracellular environment, with particular focus on cell rearrangements and dynamic switching of EC phenotype.

4. The authors mention the asymptotic value of mixing measure as 0.385. What is the physical interpretation of this value? How does this value depend on model parameters?

The asymptotic value of the mixing measure is the mean distance (normalised by the maximum possible distance in the network) that two cells, which are immediate neighbours at some fixed time, travel away from each other during 250 minutes when the vascular network has grown 'sufficiently'. Before this 'sufficient' size is reached, the mixing measure increases monotonically towards its asymptotic value as we show in our simulation results.

We have now performed an extensive sensitivity analysis of parameters of the cellular and tissue scales and investigated how the asymptotic value of the mixing measure is affected. We find that this quantity is robust to variations in model parameters. Interestingly, the majority of variations (both positive and negative) of the model parameters leads to a decrease in the asymptotic value of the mixing measure. These new results can be found in Supplementary material, S1 Text.

5. The authors have not performed a detailed sensitivity analysis. Given their framework, one of the approaches in enabling more confidence about the robustness of their predictions can be performing some principal component analysis to map combinations of input parameters to specific outputs (Fig 2; Pramanik et al. bioRxiv 2020, 041632).

We thank the reviewer for this suggestion; we have now performed an extensive sensitivity analysis. Since the subcellular scale model has been analysed in previous work [Boareto et al., 2015a], we focused our sensitivity analysis on parameters at the cellular and tissue scales. We also thank the reviewer for suggesting that we present the results of our sensitivity analysis as in [Pramanik et al., 2020]. We have used this idea in Fig 13 and S2 Fig of the revised manuscript.
The results can be found in **Results** section of the main text (subsection **Sensitivity analysis**, lines 700-748 of the revised manuscript) and Supplementary material, S1 Text.

Sensitivity analysis

To ascertain how variation in the baseline parameter values of our model affects the behaviour of the system, we have performed an extensive sensitivity analysis. Since the subcellular VEGF-Delta-Notch model has already been calibrated and validated independently [Boareto et al., 2015a], we have focused our analysis on the cellular and tissue scale parameters (see S2 Table). Briefly, we have performed our analysis by fixing all the parameters except one at their baseline values, and then vary the focal parameter by $\pm 0.1\%$, $\pm 5\%$, $\pm 10\%$, $\pm 15\%$, and $\pm 20\%$. This procedure is repeated for each of the tissue and cellular scale parameters. In order to quantify the impact of the variation of each parameter on both EC behaviour and network structure we have measured the following quantities:

- anterograde cell proportion (directionality metric);
- orientation;

- displacements;
- number of branching points per 100 μm^2 of vascular network area;
- number of vessel segments;
- vessel segment lengths.

Each of these metrics has been measured over 100 realisations of the multiscale system. For a full account of the details, see Supplementary material, S1 Text.

The results of our sensitivity analysis are summarised in Fig 13 and S2 Fig. Our analysis shows that system behaviour is robust to variations in most of the model parameters considered. This is indicated by the central cluster in Fig 13 and S2 Fig, highlighted in magenta, which represents those scenarios that exhibit very small deviations from the baseline behaviour. By contrast, variations of a small number of parameters produce significant deviation from the baseline behaviour (see Table 4, Fig 13 and S2 Fig).

**Par.**	**Description**	**Ref. equation**	**Metrics affected**	**Effect**
D_c	Threshold of level of Delta for initiation of ECM proteolysis	Eq (19)	All	Hyper-branching: D_c ↓; hypo-branching: D_c ↑
D_m	Threshold of level of Delta for initiation of BM assembly	Eq (21)	All	Hypo-branching: D_m ↓; hyper-branching: D_m ↑
K	Cell exploratoriness, i.e. controls the variance of the von Mises distribution	Eq (14)	All	Hyper-branching: K ↓; hypo-branching: K ↑
E_{F1}	Threshold of the level of cell-cell contact necessary for cell movement initiation	Eq (9)	Orientation, directionality	Hyper-branching: E_{F1} ↓
E_{F2}	Threshold of the inhibitory effect of crowding on cell movement	Eq (9)	Orientation, directionality	Hypo-branching: E_{F2} ↓

Table 8. **Sensitivity analysis: parameters producing significant deviation in system behaviour from the baseline scenario.** ↓ stands for decrease of the focal parameter, ↑ - increase of the focal parameter.

Specifically, we observe that an increase in D_m and a decrease in both D_c and K induce excessive branching, with shorter average vessel length (see Fig 13, hyper-branching region highlighted in brown). By contrast, a decrease in D_m and an increase in both D_c and K induce less branched networks, with longer average vessel length (see Fig 13, hypo-branching region highlighted in grey). These results are in agreement with well-known features of tumour vasculature, where the tumour microenvironment inhibits vessel stabilisation; specifically, it hinders the formation of the basal membrane in tumour vasculature, yielding aberrant, excessively branched, networks [Papetti and Herman, 2002]. This phenomenon can be realised by increasing D_m. Furthermore, proteolysis is also up-regulated during tumour-induced angiogenesis due to secretion of MMPs by cancer cells. Proteolysis reduces the resistance experienced by the ECs as they migrate towards the tumour [Papetti and Herman, 2002]. This effect can be accounted for phenomenologically by a reduction in D_c. Increased stimulation with growth factors that can also bind to VEGF receptors (as in pathological angiogenesis [Gupta and Qin, 2003, Papetti and Herman, 2002]) can reduce the response of the ECs to chemotactic stimuli, due to high occupancy of receptors all over the cell membrane, thus shifting cell behaviour to chemokinesis (non-directional cell migration) [Plank and Sleeman, 2003, Barkefors et al., 2008]. In our model, this transition is controlled by the cell exploratoriness, κ (see Eq (9)): for high values of κ (i.e. higher values of K) cell migration is directed along the sprout elongation vector, whereas for small values of κ (i.e. smaller values of K) cells exhibit exploratory behaviour corresponding to chemokinesis.

Our model thus predicts that changes in D_c, D_m and K are likely to occur in tumour-induced angiogenesis. This prediction is supported by current knowledge regarding the effects of the presence of a tumour in the microenvironment [Gupta and Qin, 2003, Papetti and Herman, 2002, Plank and Sleeman, 2003].

6. The authors mention about a "sweet spot" of collagen density. They should discuss their results in the context of a recent study showing optimality of cell invasion at intermediate matrix stiffness (Ahmadzadeh et al. PNAS 2017).

Cell migration was shown to be largely influenced by the surrounding extracellular environment including substrate stiffness, its density and fibre alignment. In [Ahmadzadeh et al., 2017], cell invasion into the surrounding ECM was investigated on an example of melanoma cells. It was shown that for melanoma cells, cell invasion is optimal on substrates of intermediate stiffnesses. Although matrix density changes as its stiffness varies, treatment of melanoma cells with metalloprotease MMP2 inhibitor only slightly reduced cell invasion, and the biphasic response of the cells with respect to ECM stiffness was maintained. By contrast, invasion of endothelial cells has been shown to be directly related to their proteolytic ability (metalloprotease MMP1 secretion) [Collen et al., 2003, Galvez et al., 2001, Shamloo and Heilshorn, 2010]. The results of [Shamloo and Heilshorn, 2010], which we use for our model calibration, showed that at low collagen density ECs invade the ECM freely but without forming sprouting structures. On the other hand, at high collagen densities, ECs are unable to invade the matrix and short thick protrusions were formed. Thus, the 'sweet spot' in [Shamloo and Heilshorn, 2010] is more related to ECs ability to form typical vascular network sprouting structures than cell invasion, as was the main focus of [Ahmadzadeh et al., 2017]. This has been clarified in lines 646-649 of the revised manuscript:

Furthermore, we note that the "sweet spot" of ECM concentration is related to EC ability to form typical angiogenic sprouting structures rather than to their ability to invade the ECM which decreases as the ECM concentration, c_{max}, increases (see Fig 11).

7. Can the authors make an effort to connect their predictions to latest observations about dynamics of angiogenesis, such as in Kang et al. PNAS 2019, Antfolk et al. PNAS 2017?

We thank the reviewer for these references. Both articles focus on the subcellular signalling of ECs and how this relates to phenotype selection. As we have already mentioned, the main goal of our work is not to investigate in greater detail the intracellular signalling pathways but to develop a model that connects cell migration with cell phenotype and the extracellular environment. Extending the subcellular scale model is a direction of our future work. We have included these references in lines 883-886 of the revised manuscript:

Furthermore, although we used a specific formulation for our subcellular model, the same modelling approach can be applied to a more/less detailed system (e.g. recent works [Antfolk et al., 2017, Kang et al., 2019]) as long as it reproduces typical phenotype patterning of cells within the vasculature.

Reviewer 3

1. The reviewer recommends to rearrange the composition of the paper (text and figures) to more highlight a part of "Model predictions", which will more interest the readers, especially biologists. The author may wish to relatively reduce the parts of model construction and validations to more appeal model prediction.

We thank the reviewer for the recommendation. In this manuscript, the **Materials and methods** section (which includes a summary of the model) precedes the **Results** section since we consider an understanding the formulation of the model is important for interpreting the simulation results. In view of the referee's suggestion, we have now revised the brief overview of the model formulation in lines 262-314 of the revised manuscript. This overview is sufficient for readers who prefer not to focus on the details of model formulation.

The model we develop is a two-dimensional stochastic multiscale model of migration-driven sprouting angiogenesis. Its structure is shown in Fig 1(B). Briefly, the model operates on three distinct spatial scales:

- The **subcellular scale** defines the gene expression pattern of individual cells. Since ECs contain finite number of proteins, some level of noise is always present in the system. Thus, we implement a stochastic model of the VEGF-Delta-Notch signalling pathway to describe the temporal dynamics of the number of ligands/receptors for each cell. This pathway is known to produce bistable behaviour. Cells exhibit either high Delta and VEGFR2 and low Notch levels (the tip phenotype) or low Delta and VEGFR2 and high Notch levels (the stalk phenotype). Stochasticity allows random transitions between these phenotypes which cannot be achieved in a deterministic model.
- The **cellular scale** accounts for cell migration. It is formulated as a variant of an on-lattice persistent random walk (PRW) of ECs.
- The **tissue scale** keeps track of the local ECM environment of the cells. Local ODEs track the evolution of the concentrations of the existing ECM and BM components, whereas ECM fibril alignment driven by EC movement is updated using a phenomenological model.

An illustration of the model geometry can be found in Fig 2.

In general, an EC has an arbitrary shape which depends on its cell-cell and cell-matrix focal adhesions. In our model we do not keep track of the exact cell shape and assume that cell position is known up to the position of its nucleus. Thus, when referring to a cell position, we refer to the position of its nucleus and assume that the cell has some arbitrary shape centred on the voxel containing its nucleus (see Fig 3(A)). A consequence of this approach is that, since cells can extend membrane protrusions and interact with distinct cells beyond their first neighbours, interactions between cells in our model are non-local. We introduce two interaction radii, R_s and R_c, for the subcellular and cellular scales, respectively, and assume that a cell can interact with any neighbouring cell partially overlapped by a circular neighbourhood of these interaction radii (see Fig 3(A)). In particular, at the subcellular scale trans-binding between a Delta ligand on one cell and a Notch receptor on another can occur if the distance between their cell centres is less than the interaction radius, R_s. Thus, the total amount of ligand/receptor (belonging to a neighbour/neighbours) to which a cell is exposed is proportional to the surface area of the overlap region between the circular neighbourhood and the neighbouring voxel/voxels (not necessarily first neighbours). A similar modelling technique is employed at the cellular scale with the interaction radius, R_c, which we use to account for cell-cell adhesion. These radii are not necessarily equal (although they are of the same order of magnitude) since different types of interactions are considered at each scale.

We perform our numerical simulations over time periods that are commensurate with the duration of *in vitro* experiments (hours) and for which proliferation is negligible [Arima et al., 2011, Gerhardt et al., 2003, Jakobsson et al., 2010]. For these reasons the model focuses only on the coordinated migration of ECs and assumes that proliferation occurs only at the vascular plexus [Gerhardt et al., 2003], the initial vascular bed. This effect is implemented by introducing ECs into the domain at a specific set of boundary voxels, $\mathcal{I}_{VP}$ (see Fig 2(A)).

Since the experimental data we use for model calibration and validation were extracted from experiments carried out with constant VEGF concentration supplied externally, we assume that the distribution of VEGF is maintained at a (prescribed) constant value at all times.

Interested readers will find more detail on the formulation of the model components at each spatial scale later in this section and in S1 Text.

We list the variables of our multi-scale model in Table 1.

2. The authors need to quantify the vascular morphology they simulated using some indices i.e. branching points and branch lengths to more efficiently analyze causative relationships between cell dynamics and morphological changes. This seems important to emphasise one of the merits of the model.

In addition to the quantification and comparison to experimental data carried out in our original manuscript, we have now performed further quantification of the simulated vascular networks using the following metrics:

- total network area;
- number of branching points per fixed area of vascular network;
- number of vascular segments;
- length of vessel segments.

Distributions of these metics were compared for different VEGF concentrations and cell linages and the results related to the evolution of the mixing measure. This information has been added to the manuscript in S7 Fig (with corresponding references to it in the main text).

3. The authors should make some efforts to evaluate their model prediction data by comparing biological data in the previous reports, if present. The reviewer understands that complete validation of their model prediction might be difficult at this moment, but they can discuss the validity using the similar biological data previously reported, which will provide important information to the readers.

The model developed in this work was calibrated and validated using results from biological experiments reported in [Arima et al., 2011, Sugihara et al., 2015, Shamloo and Heilshorn, 2010, Jakobsson et al., 2010]. For this revision, we have carried out an extensive sensitivity analysis and further model quantification which we hope will help clarify the biological scope of our model.
The results can be found in **Results** section of the main text (subsection **Sensitivity analysis**, lines 700-748 of the revised manuscript) and Supplementary material, S1 Text.

Sensitivity analysis

To ascertain how variation in the baseline parameter values of our model affects the behaviour of the system, we have performed an extensive sensitivity analysis. Since the subcellular VEGF-Delta-Notch model has already been calibrated and validated independently [Boareto et al., 2015a], we have focused our analysis on the cellular and tissue scale parameters (see S2 Table). Briefly, we have performed our analysis by fixing all the parameters except one at their baseline values, and then vary the focal parameter by $\pm 0.1\%$, $\pm 5\%$, $\pm 10\%$, $\pm 15\%$, and $\pm 20\%$. This procedure is repeated for each of the tissue and cellular scale parameters. In order to quantify the impact of the variation of each parameter on both EC behaviour and network structure we have measured the following quantities:

- anterograde cell proportion (directionality metric);
- orientation;
- displacements;

- number of branching points per 100 μm^2 of vascular network area;
- number of vessel segments;
- vessel segment lengths.

Each of these metrics has been measured over 100 realisations of the multiscale system. For a full account of the details, see Supplementary material, S1 Text.

The results of our sensitivity analysis are summarised in Fig 13 and S2 Fig. Our analysis shows that system behaviour is robust to variations in most of the model parameters considered. This is indicated by the central cluster in Fig 13 and S2 Fig, highlighted in magenta, which represents those scenarios that exhibit very small deviations from the baseline behaviour. By contrast, variations of a small number of parameters produce significant deviation from the baseline behaviour (see Table 4, Fig 13 and S2 Fig).

**Par.**	**Description**	**Ref. equation**	**Metrics affected**	**Effect**
D_c	Threshold of level of Delta for initiation of ECM proteolysis	Eq (19)	All	Hyper-branching: D_c ↓; hypo-branching: D_c ↑
D_m	Threshold of level of Delta for initiation of BM assembly	Eq (21)	All	Hypo-branching: D_m ↓; hyper-branching: D_m ↑
K	Cell exploratoriness, i.e. controls the variance of the von Mises distribution	Eq (14)	All	Hyper-branching: K ↓; hypo-branching: K ↑
E_{F1}	Threshold of the level of cell-cell contact necessary for cell movement initiation	Eq (9)	Orientation, directionality	Hyper-branching: E_{F1} ↓
E_{F2}	Threshold of the inhibitory effect of crowding on cell movement	Eq (9)	Orientation, directionality	Hypo-branching: E_{F2} ↓

Table 12. **Sensitivity analysis: parameters producing significant deviation in system behaviour from the baseline scenario.** ↓ stands for decrease of the focal parameter, ↑ - increase of the focal parameter.

Specifically, we observe that an increase in D_m and a decrease in both D_c and K induce excessive branching, with shorter average vessel length (see Fig 13, hyper-branching region highlighted in brown). By contrast, a decrease in D_m and an increase in both D_c and K induce less branched networks, with longer average vessel length (see Fig 13, hypo-branching region highlighted in grey). These results are in agreement with well-known features of tumour vasculature, where the tumour microenvironment inhibits vessel stabilisation; specifically, it hinders the formation of the basal membrane in tumour vasculature, yielding aberrant, excessively branched, networks [Papetti and Herman, 2002]. This phenomenon can be realised by increasing D_m. Furthermore, proteolysis is also up-regulated during tumour-induced angiogenesis due to secretion of MMPs by cancer cells. Proteolysis reduces the resistance experienced by the ECs as they migrate towards the tumour [Papetti and Herman, 2002]. This effect can be accounted for phenomenologically by a reduction in D_c. Increased stimulation with growth factors that can also bind to VEGF receptors (as in pathological angiogenesis [Gupta and Qin, 2003, Papetti and Herman, 2002]) can reduce the response of the ECs to chemotactic stimuli, due to high occupancy of receptors all over the cell membrane, thus shifting cell behaviour to chemokinesis (non-directional cell migration) [Plank and Sleeman, 2003, Barkefors et al., 2008]. In our model, this transition is controlled by the cell exploratoriness, κ (see Eq (9)): for high values of κ (i.e. higher values of K) cell migration is directed along the sprout elongation vector, whereas for small values of κ (i.e. smaller values of K) cells exhibit exploratory behaviour corresponding to chemokinesis.

Our model thus predicts that changes in D_c, D_m and K are likely to occur in tumour-induced angiogenesis. This prediction is supported by current knowledge regarding the effects of the presence of a tumour in the microenvironment [Gupta and Qin, 2003, Papetti and Herman, 2002, Plank and Sleeman, 2003].

4. Notation for VEGF receptor mutant cells, R2+ an R1+, is quite confusing to the reviewer and maybe the readers including biologists, because generally VEGFR2 wild type, heterozygote and homozygote cells are represented as VEGFR2+/+, VEGFR2+/- and VEGFR2-/-, respectively. The reviewer recommends to change the notation to others.

We thank the reviewer for this suggestion. We have revised the notation to VEGFR2$^{+/-}$ and VEGFR1$^{+/-}$ for heterozygous receptors VEGFR2 and VEGFR1, respectively, throughout text and figures.

References

Ahmadzadeh et al., 2017. Ahmadzadeh, H., Webster, M. R., Behera, R., Valencia, A. M. J., Wirtz, D., Weeraratna, A. T., and Shenoy, V. B. (2017). Modeling the two-way feedback between contractility and matrix realignment reveals a nonlinear mode of cancer cell invasion. Proceedings of the National Academy of Sciences, 114(9):E1617–E1626.

Antfolk et al., 2017. Antfolk, D., Sjöqvist, M., Cheng, F., Isoniemi, K., Duran, C. L., Rivero-Muller, A., Antila, C., Niemi, R., Landor, S., Bouten, C. V., et al. (2017). Selective regulation of notch ligands during angiogenesis is mediated by vimentin. Proceedings of the National Academy of Sciences, 114(23):E4574–E4581.

Arima et al., 2011. Arima, S., Nishiyama, K., Ko, T., Arima, Y., Hakozaki, Y., Sugihara, K., Koseki, H., Uchijima, Y., Kurihara, Y., and Kurihara, H. (2011). Angiogenic morphogenesis driven by dynamic and heterogeneous collective endothelial cell movement. Development, 138(21):4763–4776.

Barkefors et al., 2008. Barkefors, I., Le Jan, S., Jakobsson, L., Hejll, E., Carlson, G., Johansson, H., Jarvius, J., Park, J. W., Jeon, N. L., and Kreuger, J. (2008). Endothelial cell migration in stable gradients of vascular endothelial growth factor a and fibroblast growth factor 2 effects on chemotaxis and chemokinesis. Journal of Biological Chemistry, 283(20):13905–13912.

Bentley et al., 2014. Bentley, K., Franco, C. A., Philippides, A., Blanco, R., Dierkes, M., Gebala, V., Stanchi, F., Jones, M., Aspalter, I. M., Cagna, G., Westrom, S., Claesson-Welsh, L., Vestweber, D., and Gerhardt, H. (2014). The role of differential ve-cadherin dynamics in cell rearrangement during angiogenesis. Nature Cell Biology, 16(309):309–321.

Bentley et al., 2009. Bentley, K., Mariggi, G., Gerhardt, H., and Bates, P. A. (2009). Tipping the balance: Robustness of tip cell selection, migration and fusion in angiogenesis. PLOS Computational Biology, 5:1–19.

Boareto et al., 2015a. Boareto, M., Jolly, M. K., Ben-Jacob, E., and Onuchic, J. N. (2015a). Jagged mediates differences in normal and tumor angiogenesis by affecting tip-stalk fate decision. Proceedings of the National Academy of Sciences, 112(29):E3836–E3844.

Boareto et al., 2015b. Boareto, M., Jolly, M. K., Lu, M., Onuchic, J. N., Clementi, C., and Ben-Jacob, E. (2015b). Jagged–delta asymmetry in notch signaling can give rise to a sender/receiver hybrid phenotype. Proceedings of the National Academy of Sciences, 112(5):E402–E409.

Boas and Merks, 2015. Boas, S. E. M. and Merks, R. M. H. (2015). Tip cell overtaking occurs as a side effect of sprouting in computational models of angiogenesis. BMC Systems Biology, 9(1):86.

Collen et al., 2003. Collen, A., Hanemaaijer, R., Lupu, F., Quax, P. H., van Lent, N., Grimbergen, J., Peters, E., Koolwijk, P., and van Hinsbergh, V. W. (2003). Membrane-type matrix metalloproteinase-mediated angiogenesis in a fibrin-collagen matrix. Blood, The Journal of the American Society of Hematology, 101(5):1810–1817.

Galvez et al., 2001. Galvez, B. G., Matias, Roman, S., Albar, J. P., Sanchez-Madrid, F., and Arroyo, A. G. (2001). Membrane type 1-matrix metalloproteinase is activated during migration of human endothelial cells and modulates endothelial motility and matrix remodeling. Journal of Biological Chemistry, 276(40):37491–37500.

Gerhardt et al., 2003. Gerhardt, H., Golding, M., Fruttiger, M., Ruhrberg, C., Lundkvist, A., Abramsson, A., Jeltsch, M., Mitchell, C., Alitalo, K., Shima, D., et al. (2003). Vegf guides angiogenic sprouting utilizing endothelial tip cell filopodia. The Journal of Cell Biology, 161(6):1163–1177.

Giniūnaitė et al., 2020. Giniūnaitė, R., Baker, R. E., Kulesa, P. M., and Maini, P. K. (2020). Modelling collective cell migration: neural crest as a model paradigm. Journal of Mathematical Biology, 80(1):481–504.

Gupta and Qin, 2003. Gupta, M. K. and Qin, R.-Y. (2003). Mechanism and its regulation of tumor-induced angiogenesis. World journal of gastroenterology: WJG, 9(6):1144.

Jakobsson et al., 2010. Jakobsson, L., Franco, C. A., Bentley, K., Collins, R. T., Ponsioen, B., Aspalter, I. M., Rosewell, I., Busse, M., Thurston, G., Medvinsky, A., et al. (2010). Endothelial cells dynamically compete for the tip cell position during angiogenic sprouting. Nature Cell Biology, 12(10):943.

Kang et al., 2019. Kang, T.-Y., Bocci, F., Jolly, M. K., Levine, H., Onuchic, J. N., and Levchenko, A. (2019). Pericytes enable effective angiogenesis in the presence of proinflammatory signals. Proceedings of the National Academy of Sciences, 116(47):23551–23561.

Koon et al., 2018. Koon, Y. L., Zhang, S., Rahmat, M. B., Koh, C. G., and Chiam, K.-H. (2018). Enhanced delta-notch lateral inhibition model incorporating intracellular notch heterogeneity and tension-dependent rate of delta-notch binding that reproduces sprouting angiogenesis patterns. Scientific reports, 8(1):1–15.

Lamalice et al., 2007. Lamalice, L., Le Boeuf, F., and Huot, J. (2007). Endothelial cell migration during angiogenesis. Circulation Research, 100(6):782–794.

Papetti and Herman, 2002. Papetti, M. and Herman, I. M. (2002). Mechanisms of normal and tumor-derived angiogenesis. American Journal of Physiology-Cell Physiology, 282(5):C947–C970.

Plank and Sleeman, 2003. Plank, M. and Sleeman, B. (2003). Tumour-induced angiogenesis: a review. Journal of Theoretical Medicine, 5(3-4):137–153.

Potente and Carmeliet, 2017. Potente, M. and Carmeliet, P. (2017). The link between angiogenesis and endothelial metabolism. Annual review of physiology, 79:43–66.

Pramanik et al., 2020. Pramanik, D., Jolly, M. K., and Bhat, R. (2020). Interactive dynamics of matrix adhesion and reaction-diffusion predict diverse multiscale strategies of cancer cell invasion. bioRxiv.

Reinhart-King et al., 2005. Reinhart-King, C. A., Dembo, M., and Hammer, D. A. (2005). The dynamics and mechanics of endothelial cell spreading. Biophysical Journal, 89(1):676–689.

Rousseau et al., 2000. Rousseau, S., Houle, F., Kotanides, H., Witte, L., Waltenberger, J., Landry, J., and Huot, J. (2000). Vascular endothelial growth factor (vegf)-driven actin-based motility is mediated by vegfr2 and requires concerted activation of stress-activated protein kinase 2 (sapk2/p38) and geldanamycin-sensitive phosphorylation of focal adhesion kinase. Journal of Biological Chemistry, 275(14):10661–10672.

Shamloo and Heilshorn, 2010. Shamloo, A. and Heilshorn, S. C. (2010). Matrix density mediates polarization and lumen formation of endothelial sprouts in vegf gradients. Lab on a Chip, 10(22):3061–3068.

Sprinzak et al., 2010. Sprinzak, D., Lakhanpal, A., LeBon, L., Santat, L. A., Fontes, M. E., Anderson, G. A., Garcia-Ojalvo, J., and Elowitz, M. B. (2010). Cis-interactions between notch and delta generate mutually exclusive signalling states. Nature, 465(7294):86.

Sugihara et al., 2015. Sugihara, K., Nishiyama, K., Fukuhara, S., Uemura, A., Arima, S., Kobayashi, R., Köhn-Luque, A., Mochizuki, N., Suda, T., Ogawa, H., et al. (2015). Autonomy and non-autonomy of angiogenic cell movements revealed by experiment-driven mathematical modeling. Cell Reports, 13(9):1814–1827.

Szabó and Mayor, 2016. Szabó, A. and Mayor, R. (2016). Modelling collective cell migration of neural crest. Current opinion in cell biology, 42:22–28.

Theveneau and Mayor, 2013. Theveneau, E. and Mayor, R. (2013). Collective cell migration of epithelial and mesenchymal cells. Cellular and Molecular Life Sciences, 70(19):3481–3492.

Vega et al., 2020. Vega, R., Carretero, M., Travasso, R. D., and Bonilla, L. L. (2020). Notch signaling and taxis mechanims regulate early stage angiogenesis: A mathematical and computational model. PLoS computational biology, 16(1):e1006919.

Watanabe et al., 2005. Watanabe, T., Noritake, J., and Kaibuchi, K. (2005). Regulation of microtubules in cell migration. Trends in cell biology, 15(2):76–83.

Zhang et al., 2019. Zhang, J., Goliwas, K. F., Wang, W., Taufalele, P. V., Bordeleau, F., and Reinhart-King, C. A. (2019). Energetic regulation of coordinated leader–follower dynamics during collective invasion of breast cancer cells. Proceedings of the National Academy of Sciences, 116(16):7867–7872.
